# Hymenopteran parasitoids reared from European gall midges (Diptera, Cecidomyiidae)

**DOI:** 10.3897/BDJ.12.e118487

**Published:** 2024-03-25

**Authors:** Hans Henrik Bruun, Simon Haarder, Peter Neerup Buhl, Richard R. Askew

**Affiliations:** 1 University of Copenhagen, Copenhagen, Denmark University of Copenhagen Copenhagen Denmark; 2 private researcher, Vordingborg, Denmark private researcher Vordingborg Denmark; 3 private researcher, Sakskøbing, Denmark private researcher Sakskøbing Denmark; 4 private researcher, St Marcel du Perigord, France private researcher St Marcel du Perigord France

**Keywords:** Cecidomyiidae, host-parasitoid interactions, Eltonian shortfall, rearing methods, Ceraphronidae, Encyrtidae, Eulophidae, Eupelmidae, Eurytomidae, Pteromalidae, Torymidae, Platygastridae

## Abstract

We report the results of investigations 2010 through 2023 of hymenopteran parasitoids associated with gall midges in Europe. A total of 242 collections of gall midges were made, from each of which one to several parasitoid species emerged, resulting in ca. 200 recorded parasitoid species and 267 host-parasitoid interaction records. The parasitoid families involved were Eulophidae (63 species), Platygastridae (56 species), Torymidae (34 species), Pteromalidae (31 species), Ceraphronidae (5 species), Eupelmidae (4 species), Eurytomidae (2 species) and Encyrtidae (1 species). As many as 159 interactions are reported for the first time, significantly enlarging our knowledge of gall midge – parasitoid interactions on the species level. Even more interesting, 51 host records are for parasitoid species for which no host was previously known. Similarly, 28 species of gall midge are reported as host to named parasitoids for the first time. Additionally, 91 parasitoid records were the first for the country in question. Differences between the rearing methods applied and their suitability for recording species with contrasting life histories, are discussed.

## Introduction

The gall midges (Diptera, Cecidomyiidae) are arguably the largest and most diverse insect family and, simultaneously, one of the most incompletely known in terms of both basic species cataloguing and knowledge of species’ biology (e.g. Espírito-Santo and Fernandes 2007, Hebert et al. 2016, Chimeno et al. 2022, Srivathsan et al. 2023). The families of parasitoid Hymenoptera associated with gall midges remain similarly very poorly known. Srivathsan et al. (2023) identified 20 insect families that account for more than half of Malaise-trapped insect species richness at sites across the globe. Cecidomyiidae and two families of parasitoid Hymenoptera frequently associated with them, i.e. Platygastridae and Eulophidae, figured prominently in the list. The large proportion of undescribed species, “dark taxa”, highlights the shortfall in current biodiversity knowledge. Another equally important knowledge gap is the ignorance of species’ trophic interactions, something that has been coined the “Eltonian shortfall” (Hortal et al. 2015).

The Cecidomyiidae are small delicate flies (Tokuda and Yukawa 2021). The majority of species are phytophagous and display a wide range of host-plant relationships and life-history strategies (Dorchin et al. 2019). Many species induce galls on the host plants, but others inhabit leaf sheaths, grass spikelets or dense inflorescences without causing obvious malformation of the plant parts. Many species are univoltine, fewer are bivoltine or multivoltine. In most temperate species, larvae leave their host plant to hibernate and later pupate in the soil. Pupation in situ, for example, in galls, seems to have arisen several times within the clade. All of these life-history characteristics have been shown to influence the size and family composition of their associated parasitoid assemblages (Hawkins and Gagné 1989).

The study of gall midge parasitoids is almost as old as studies of the gall midges themselves. For example, J.-J. Kieffer (1857-1925) described several hundred species of Cecidomyiidae and Platygastridae and some Chalcidoidea. H. F. Barnes (1902-1960) and his students made numerous biological studies on gall midges and their parasitoids, in particular, species associated with crops and ornamental plants. More recently, Skuhravá and Thuróczy (2007) reported 39 species of Chalcidoidea and Platygastroidea reared from galls of 50 species of gall midges, which had been collected between 1955 and 1996 in the Czech Republic and elsewhere in central and southern Europe. Similar studies have been published by Tudor and Neacşu (1983), Askew and Harris (2007), Buhl and Jørgensen (2010), Jennings (2021) and others. Nevertheless, the knowledge of gall midge - parasitoid relationships remains incomplete at best and is mainly based on rearing adult parasitoids directly from midge-induced galls.

The Chalcidoidea parasitoids of gall midges are mainly found in the families Eulophidae, Pteromalidae (incl. Pirenidae and Systasidae) and Torymidae, with fewer species of Braconidae, Encyrtidae and Eurytomidae being involved (Yukawa et al. 2021). Another major group of gall midge parasitoids is Platygastridae (Platygastroidea), while fewer Ceraphronidae (Ceraphronoidea) have been recorded. This suite of hymenopteran parasitoids exhibits a wide and complex array of life-history characteristics. One helpful simplification is the division of parasitoids into koinobionts and idiobionts (sensu Askew and Shaw (1986)). Koinobionts let their hosts continue normal development and behaviour, such as the midge larvae leaving the galls to continue life and to pupate elsewhere. Koinobionts are usually endoparasitoids and expected to be more narrowly host-specialised, due to the prolonged physiological intimacy with a living and metabolising host (Hawkins 1994). Idiobionts, in contrast, immediately kill or permanently paralyse their hosts. They are typically ectoparasitoids and they sometimes show association with particular host plants, attacking phylogenetically diverse assemblages of phytophagous insects using that particular host plant (Hawkins 1994).

Here, we report primary data on the parasitoid Hymenoptera that, during the years 2010 – 2023, we have reared from larvae of Cecidomyiidae in Europe using a suite of methods. The methodology is discussed in relation to the life-history characteristics of both gall midge hosts and of their koinobiont and idiobiont parasitoids.

## Materials and methods

Gall midge larvae of a suite of species were collected from their natural feeding site, be it plant galls, plant inflorescences or rust (Pucciniales) sori on plants. The majority of collections were made in Denmark, several from Sweden and Poland and few from other European countries (Spain, Hungary, Romania and Lithuania). Some collections were specifically bred out to obtain adult parasitoid wasps. Other collections were made with the aim of rearing adult gall midges for taxonomic work and studying their life cycles, the parasitoids being obtained as a bycatch. Thus, the collection strategy may be considered opportunistic, dictated by the species of gall midges that we have happened to encounter.

Methods for obtaining adult parasitoid hymenoptera:

1. Adults extracted from galls (1A) or collected while ovipositing on midge-inhabited galls (1B);
2. Adults emerging directly from galls or other plants parts inhabited by gall midge larvae, sometimes from galls kept over winter;
3. Adults emerging from mature larvae or pupae extracted from galls and transferred individually to gelatine capsules;
4. Adults emerging from soil in pots, to which gall midge larvae had been transferred earlier the same year (4A) or in the preceding year (4B). Parasitoid emergence is often simultaneous with adult gall midge emergence, but is sometimes delayed (even up to a year);
5. No rearing attempted, or rearing failed; parasitoid reported at the genus level if possible.

Method 4 was applied to gall midge species which hibernate and pupate in the soil. Midge larvae were either transferred to clean soil in pots using a small paintbrush or, in some cases, the inhabited plant parts were left on the soil surface in similar pots for a few days (then removed to avoid mould), allowing the larvae to move to the soil of their own accord. Often, a combination of the two ways was used, first an appreciable number of larvae were transferred by hand to ensure a minimum number were secured, and then additional inhabited plant parts were added. Larvae of endoparasitic, koinobiont Hymenoptera would be transferred with the midge larvae, while ectoparasitic larvae destined to pupate in situ may potentially have been discarded with the plant parts. Pots with inhabited soil were kept overwinter outdoors in Kårup Skov, Denmark under ambient conditions of temperature and precipitation until early the following spring, then taken indoors to allow adult insects to emerge from pots in mesh bags or spacious perforated plastic bags.

The identification work was based on available keys, original diagnoses, revisions and comparision with paratypes and other specimens kept in the personal collections of RRA and PNB. With regard to the many Platygastridae originally described by Walker, we based our work on the revision and re-description of species by Vlug (1985). We report all parasitoid records that can be considered new to the given gall midge host, even in the many cases where the parasitoid identification is incomplete and requires more work, including rearing more material, undertaking taxonomic revisions and describing new species. That choice is motivated by our focus on the “Eltonian shortfall”.

## Results

A total of 242 collections was made, representing 109 Cecidomyiidae species, some of which belong to species that are yet to be formally described (designated an interim name to avoid ambiguity).

A number of gall midge samples were reared without yielding any parasitoids, despite quite plentiful material (> 100 of adult midges emerged). These species include *Coniophoragraminicola* Nijveldt, 1959, *Dasineurairregularis* (Bremi, 1847), *Dasineurasisymbrii* (Schrank, 1803), *Jaapiellachelidonii* Fedotova 2008 and *Wachtliellacaricis* (Loew, 1850). Clearly, these observations may represent nothing more than chance absence of parasitoids in single gall midge populations.

**Figure 1.**
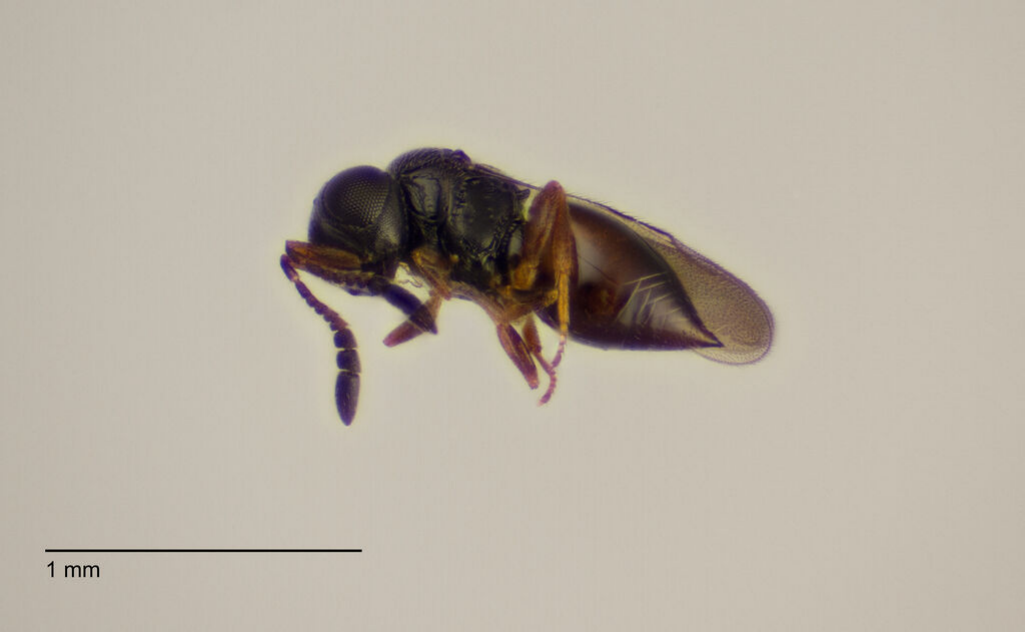
Family Ceraphronidae. Adult female *Aphanogmusabdominalis* emerged with its host *Dasineuraodoratae* from galls on *Violaodorata*. Ornebjerg, Denmark. Photo: Simon Haarder.

**Figure 3.**
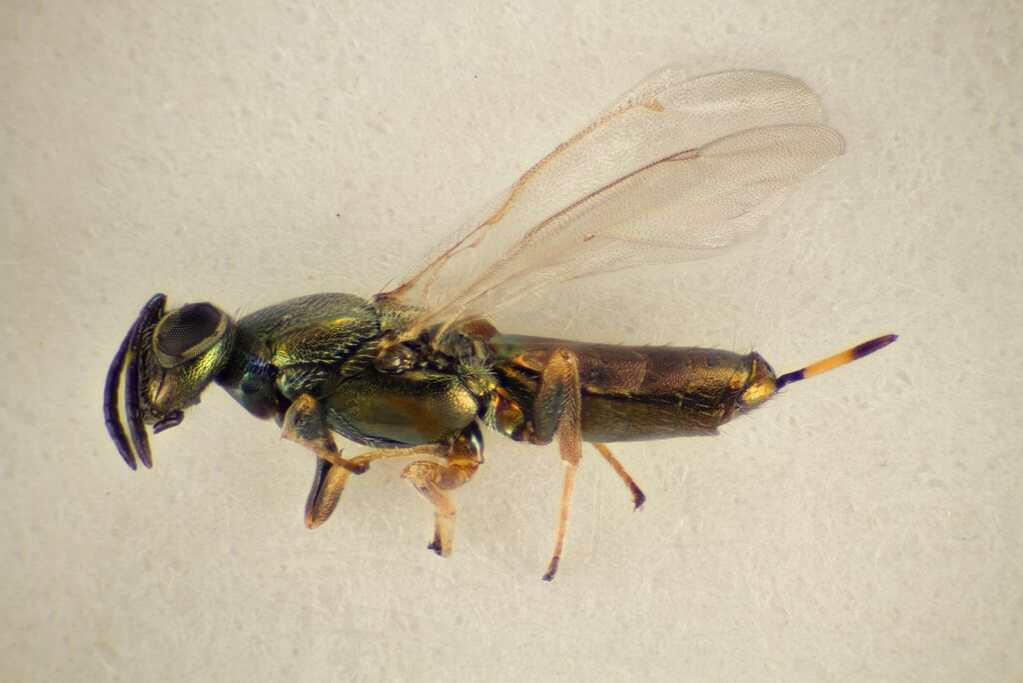
Family Eupelmidae. Adult female *Eupelmusconfusus*, an idiobiont ectoparasitoid of *Asphondyliasarothamni*, summer generation gall on *Cytisusscoparius*. Hårbølle Havn, Denmark. Photo: Simon Haarder.

**Figure 4.**
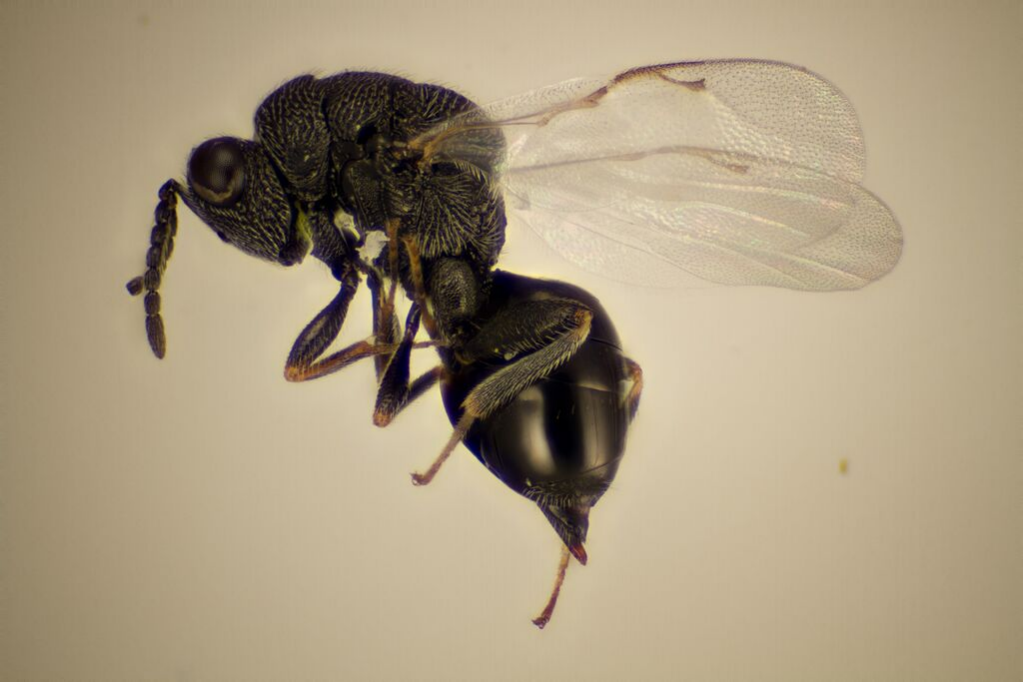
Family Eurytomidae. Adult female *Eurytomadentata*, an idiobiont ectoparasitoid of *Asphondyliasarothmni*, summer generation gall on *Cytisusscoparius*. Ornebjerg, Denmark. Photo: Simon Haarder.

Approximately 200 parasitoid taxa emerged, belonging to eight Hymenoptera families, i.e. Ceraphronidae (5 species, Fig. 1), Encyrtidae (1 species), Eulophidae (63 species, Fig. 2), Eupelmidae (4 species, Fig. 3), Eurytomidae (2 species, Fig. 4), Platygastridae (56 species, Fig. 5), Pteromalidae (31 species, Fig. 6) and Torymidae (34 species, Fig. 7). The figures given must be regarded as approximate because certain identification to known species was far from always possible. The full results are presented in Table 1 (with record details in Suppl. material 1). In the Table, the degree of certainty in identification to species level is indicated as follows: 1. Identified to the genus level (in one case family level), but not identified further, for example, “*Aprostocetus* sp.”; 2. Positively placed in a named species group, for example, “*Acerotella* sp. (*evanescens* group)”; 3. Morphologically close to a named taxon, but probably a separate species, for example, “Synopeassp. nrinerme”; 4. Probably the mentioned species, but confirmatory work, such as comparison to type material, is required, “Aprostocetuscf.suevius”; 5. Identified to species beyond reasonable doubt. Only identifications at level 3 to 5 were included in statistics of new host-parasitoid relations and significant biogeographic records, unless the finding represented the first record for a gall midge host of the relevant parasitoid taxon.

Of the 267 reported host-parasitoid interactions, more than half (i.e. 159) represent new host-parasitoid interaction records. Of these, 51 records make up the first host interaction for the involved parasitoid species. With 30 first host records, our method 4 (breeding out parasitoids from gall midge larvae transferred to soil to hibernate and later emerge) was particularly productive in yielding insights into the fundamental biology of the studied parasitoid species. Of the new interaction records, 28 constitute the first parasitoid record of the involved gall midge host.

Several (i.e. 85) of the records represents the first reported occurrence of the focal species in the relevant country, of which the majority (i.e. 69) refer to Denmark (Table 1).

The examined specimens are currently kept in the private collections of PNB, RRA and SH, but it is intended to place voucher specimens of the Chalcidoidea in the Natural History Museum (London) and of Ceraphronidae and Platygastridae in the Natural History Museum of Denmark (Copenhagen). The occurrence data have been uploaded to GBIF: https://doi.org/10.15468/45crz3.

## Discussion

Biotic interactions between species is an essential component of biodiversity (Gaüzère et al. 2022), but this element remains elusive as long as basic natural history knowledge on host-parasitoid relationships is incomplete or absent. Our results clearly demonstrate how incomplete our knowledge of gall midge – parasitoid interactions is. They also show that parasitoid specimens may be obtained in reasonable quantities using simple methods. These could easily be applied in school teaching and citizen science. The identification work, in contrast, requires highly specialised knowledge and remains a severe bottleneck.

With few exceptions, our host gall midge collection had low levels of replication, with most species being represented by a single collection. Thus, we expect that more species of parasitoid per gall midge species – and, conversely, more hosts per hymenopteran parasitoid - would have resulted, had our sampling allowed collection of larger quantities of hosts from more geographically dispersed sites and – in particular for gall midge species with more than one generation per year – more dispersed over the year and also between years. Previous analyses have shown plant galling Diptera, including Cecidomyiidae, to be generally associated with a low number of parasitoids per host species (Hawkins 1994). However, detailed studies of parasitoid complexes associated with individual gall midge species or groups of species using a single host plant, have demonstrated that the parasitoid communities typically consist of a dozen or more species (e.g. Tscharntke et al. (1991), Roskam (2013)). Better coverage per host species would also establish primary host-parasitoid relationships with greater certainty.

Previous analyses of the parasitoid communities associated with gall midges have found idiobionts to dominate over koinobionts by a factor two to four (Hawkins 1994, table 4.1). Our results seem to suggest a more even balance between koinobionts and idiobionts. For example, Platygastridae, which attack eggs and young larvae of gall midges (Askew 1975), are exclusively endoparasitic and koinobiont and stand out as one of the two most speciose parasitoid families, only outnumbered by Eulophidae. At the opposite end of the continuum is Torymidae subfamily Toryminae, in which the majority of species are idiobiont ectoparasitoids of insect inhabitants of plant galls, mainly attacking third instar larvae or pupae of gall midges. Pteromalidae, Eulophidae and Eurytomidae appear to take a position intermediate between these two extremes (Yukawa et al. 2021).

Torymidae were mainly obtained by our rearing method 3, i.e. extraction of mature parasitoid larvae or pupae by dissecting galls and rearing adults in gelatine capsules. In contrast, Platygastridae were mainly obtained through our method 4, i.e. breeding adult parasitoids from soil, to which host larvae had been transferred earlier. These patterns were to be expected from the life history of the parasitoids and their gall midge hosts. For Eulophidae and Pteromalidae, the two methods were about equally productive. These results suggest that more complete knowledge of parasitoid faunas of Cecidomyiidae are best obtained by a combination of rearing methods. The advantage of the first mentioned method is that it is targeted and establishes host-parasitoid relationships with greater certainty, while its disadvantage is that endoparasitoids are overlooked. Our method 2, i.e. rearing parasitoids directly from galls, the method used by far the most frequently in the past, involves the risk of obtaining parasitoids associated not with gall midges, but with other insects inhabiting the galled plant parts. Additionally, this method targets gall midge species that pupate in the galls. The main advantage of method 4 is that it is suitable for obtaining the mainly koinobiont parasitoids of gall midges leaving the galls as larvae to pupate in the soil, which is the most common condition amongst temperate Cecidomyiidae. If midge larvae are transferred to soil individually, rather than by placing inhabited plant parts on the soil surface for larvae to move voluntarily, this method is as accurate in establishing host-parasitoid relationships as is method 3. The fact that most of the first host records for parasitoid species and first parasitoid records for gall midge hosts were obtained using method 4, i.e. transfer of larvae to soil, is because this method has rarely been applied in the past. It suggests that this method is indispensable for obtaining primary life history data for koinobiont parasitoid species and for filling the knowledge gap of gall midge–parasitoid interaction networks.

## Supplementary Material

10BAA9DF-C2EC-5376-B48E-EE46104F2265 10.3897/BDJ.12.e118487.suppl1 Supplementary material 1 Supplementary data table Data type Host-parasitoid interactions Brief description Records of hymenopteran parasitoids associated with European gall midges (Diptera, Cecidomyiidae). File: oo_997102.xlsx https://binary.pensoft.net/file/997102 Hans Henrik Bruun, Simon Haarder, Peter Neerup Buhl, Richard R. Askew

## Figures and Tables

**Figure 2a.**
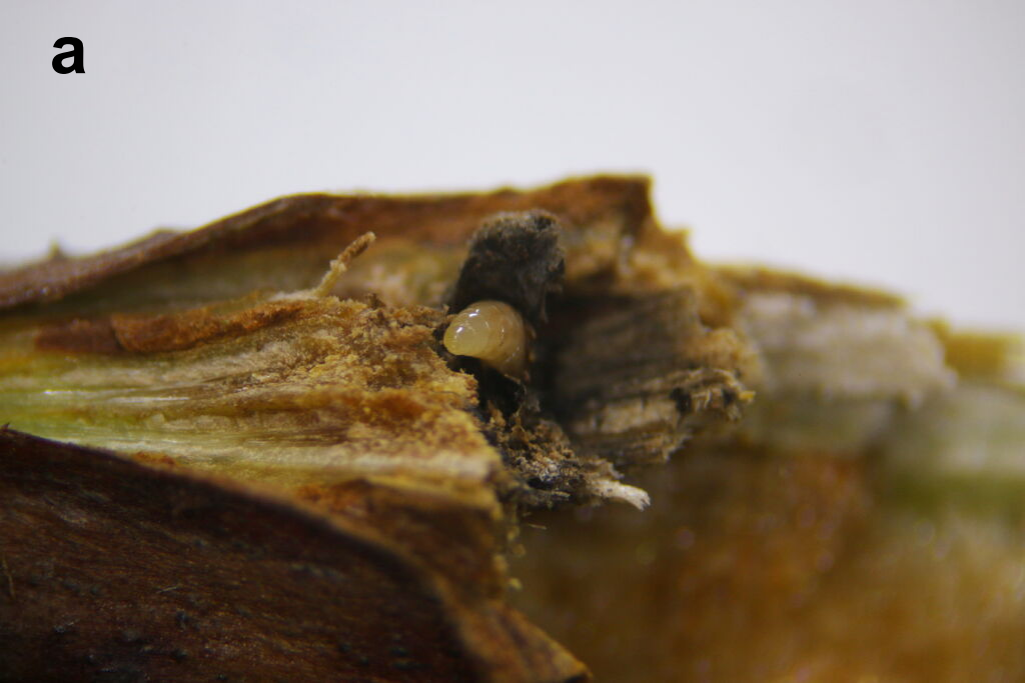
Larva of *Aprostocetusrubicola* (Eulophidae subfamily Tetrastichinae) in situ in gall of *Lasiopterarubi* in *Rubusidaeus* cane. Horreby Lyng, Denmark.

**Figure 2b.**
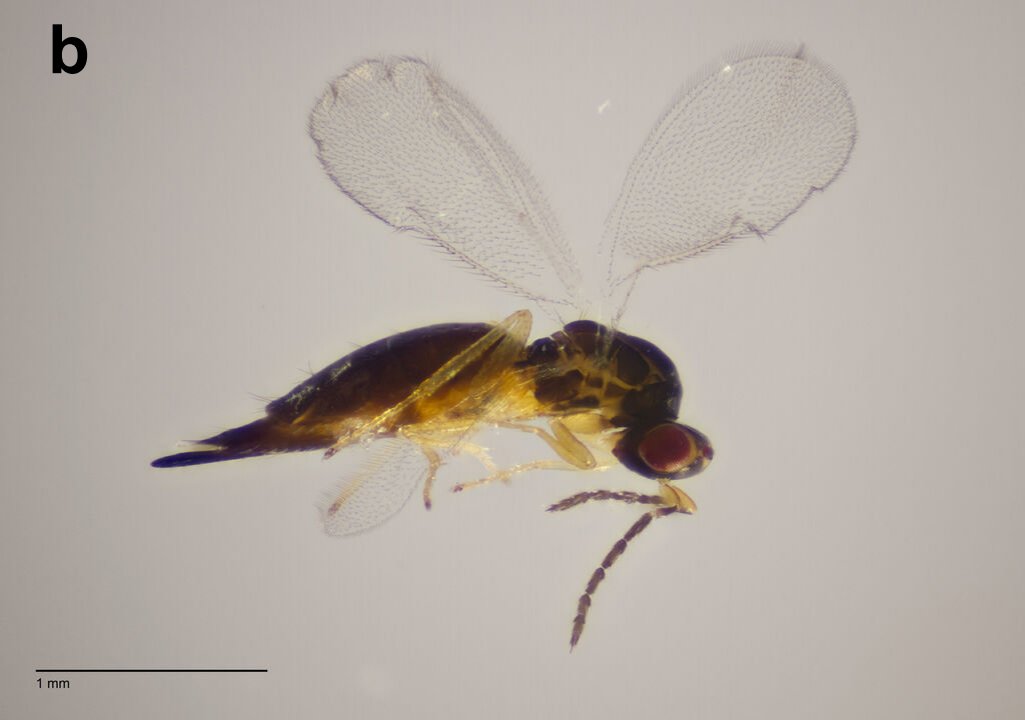
Adult female *Aprostocetusrubi*, reared using method 3. Horreby Lyng, Denmark.

**Figure 2c.**
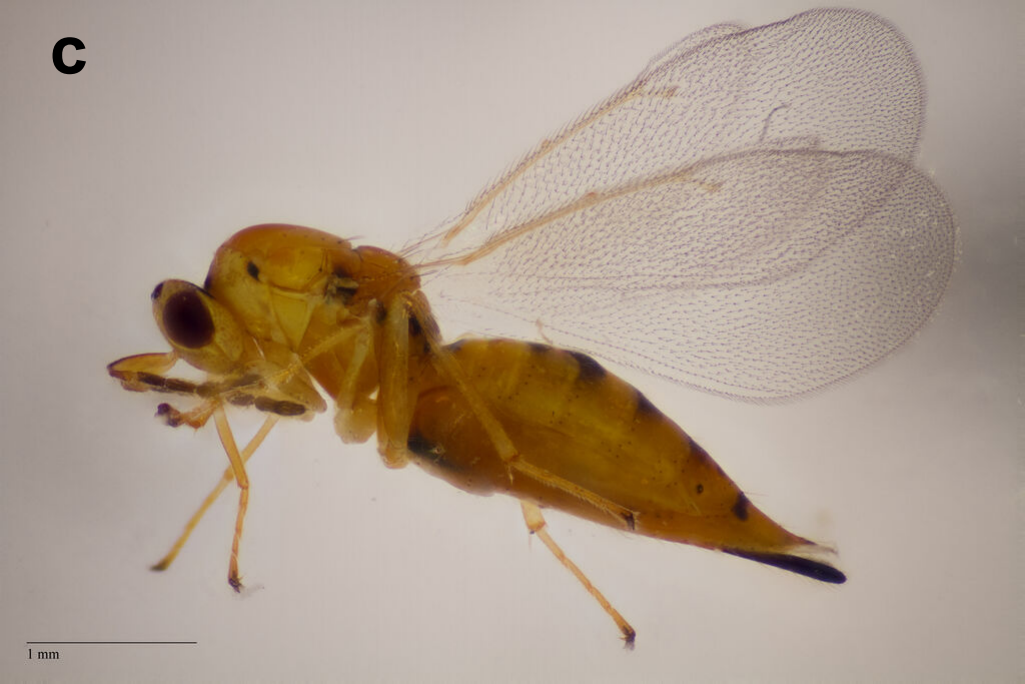
Adult female *Aprostocetusluteus*, an idiobiont ectoparasitoid of *Mikiolafagi* galling *Fagussylvatica*, reared using method 3. Ornebjerg, Denmark.

**Figure 2d.**
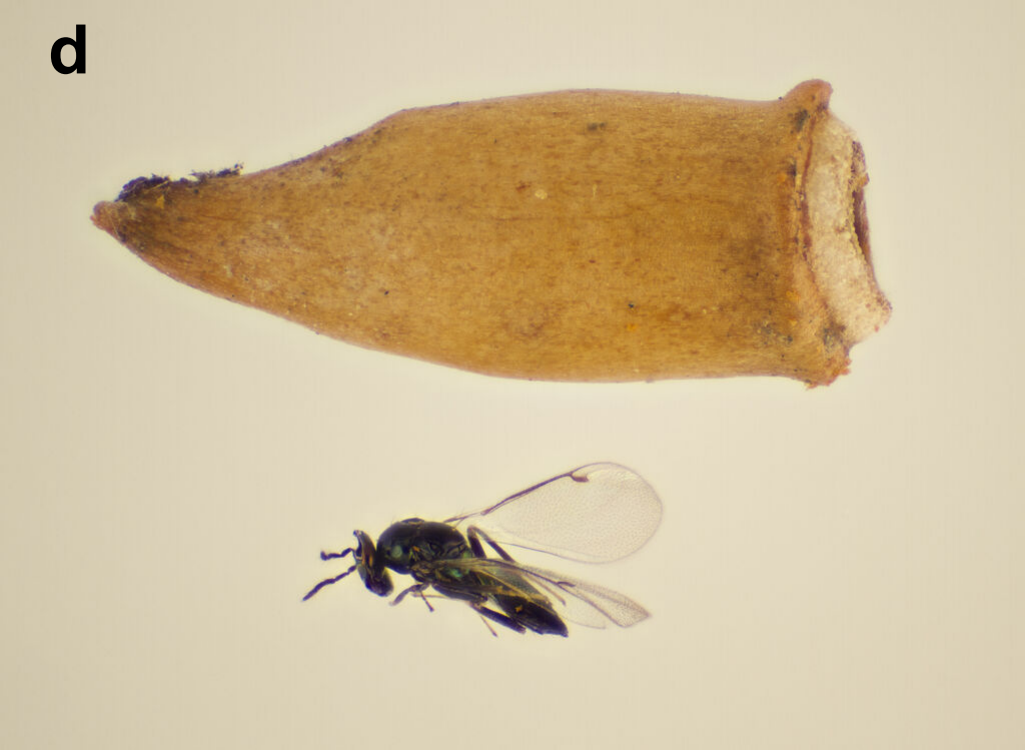
Adult female *Omphalelugens* (Eulophidae subfamily Entedoninae) with the *Mikiolafagi* gall on *Fagussylvatica*, from which it was reared. Valby, Denmark.

**Figure 5a.**
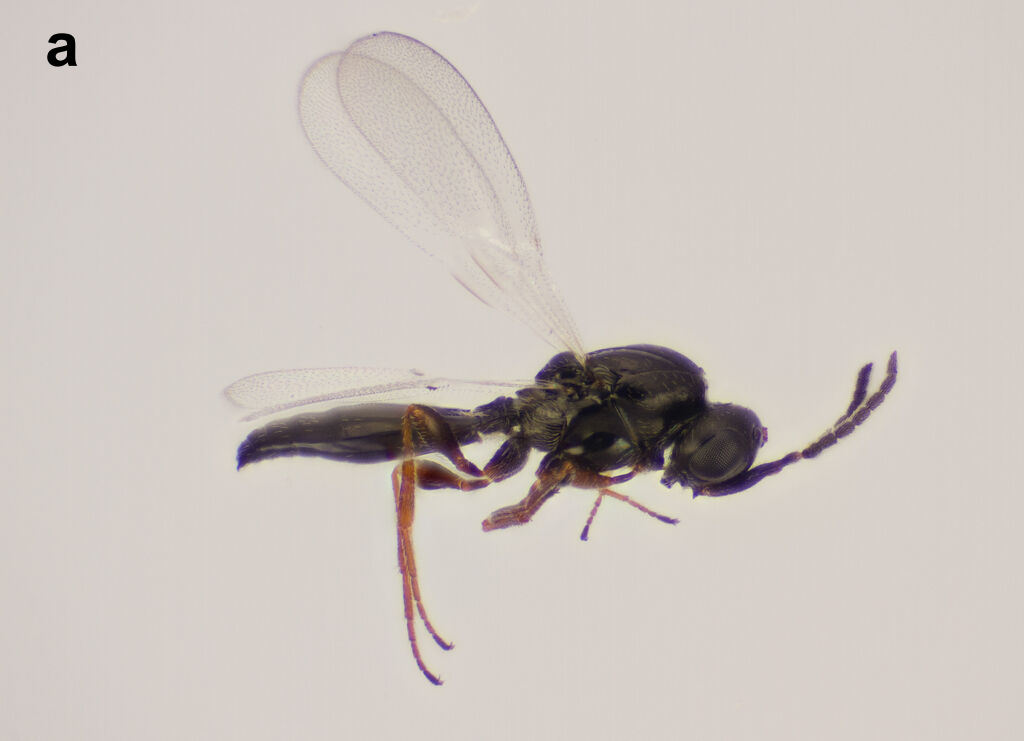
Adult male *Platygasterphragmitiphila* (Platygastridae subfamily Platygastrinae) emerged directly from galls of *Lasiopteraarundinis* on *Phragmitesaustralis*. Warszawa, Poland.

**Figure 5b.**
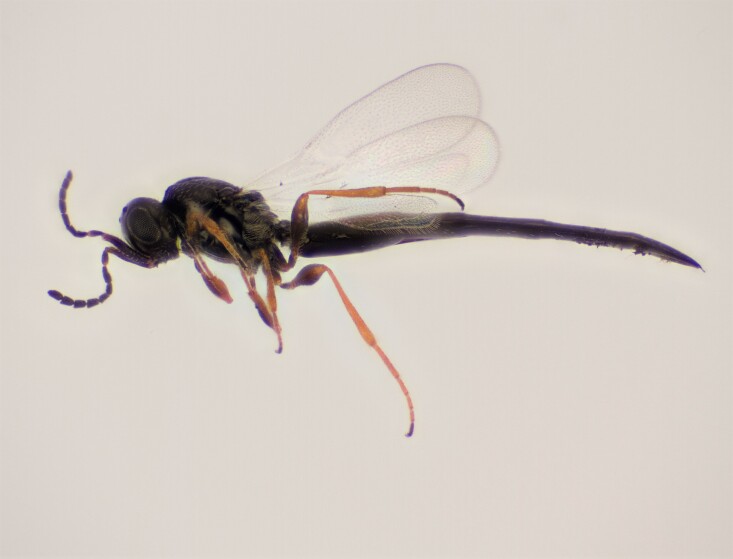
Adult female of the same collection.

**Figure 5c.**
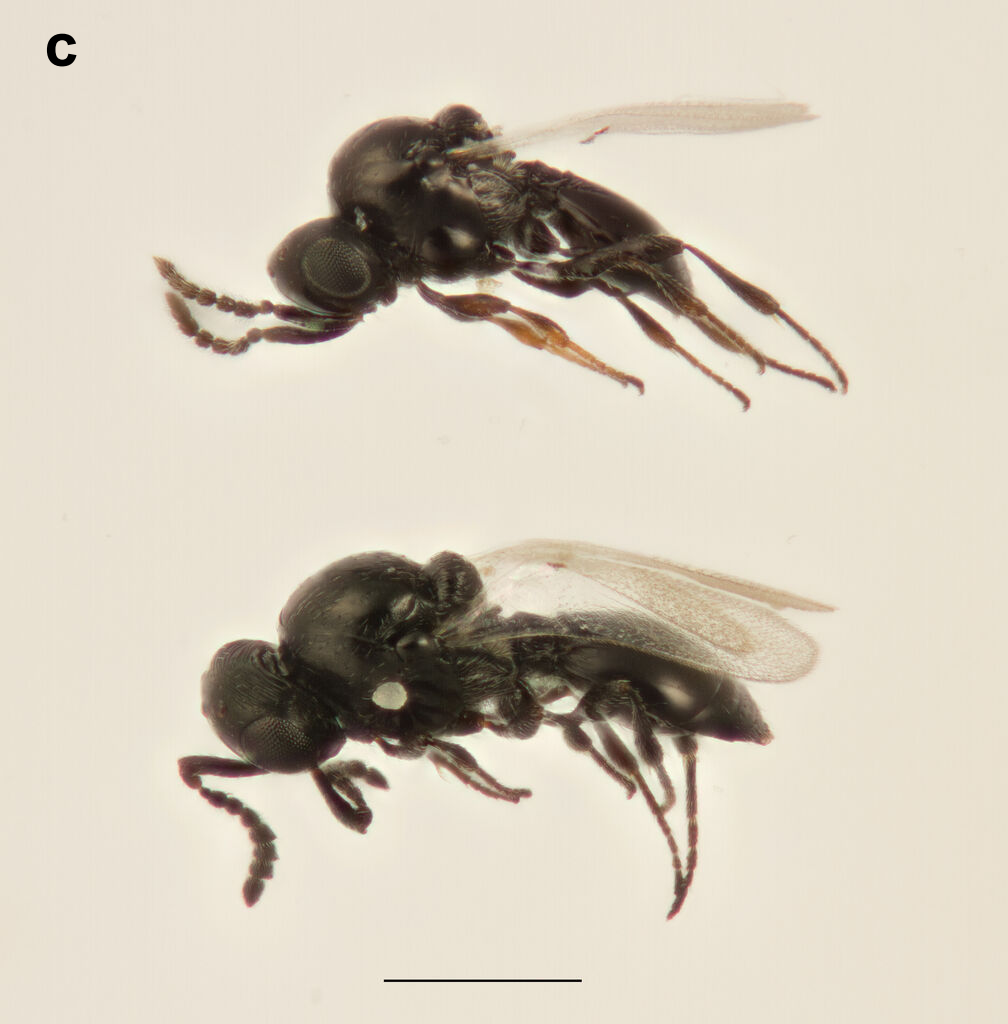
Adults of *Platygasterrobiniae*, a polyembryonal koinobiont endoparasitoid of *Obolodiplosisrobiniae*, scale bar 0.5 mm. Nykøbing Sjælland, Denmark.

**Figure 5d.**
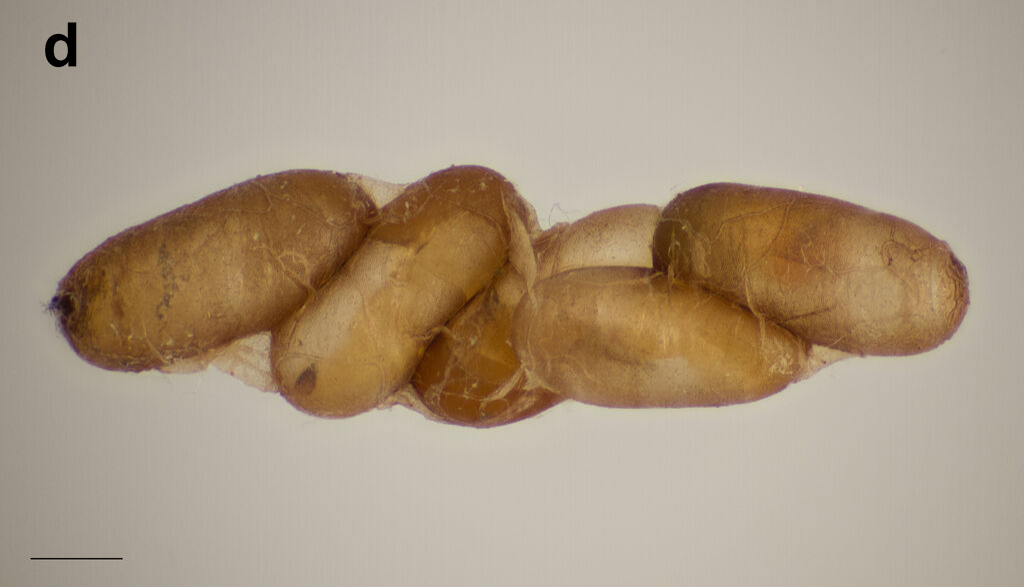
Cluster of *Platygasterrobiniae* pupae within the puparial skin of one host individual, scale bar 0.5 mm. Oxie, Sweden.

**Figure 6a.**
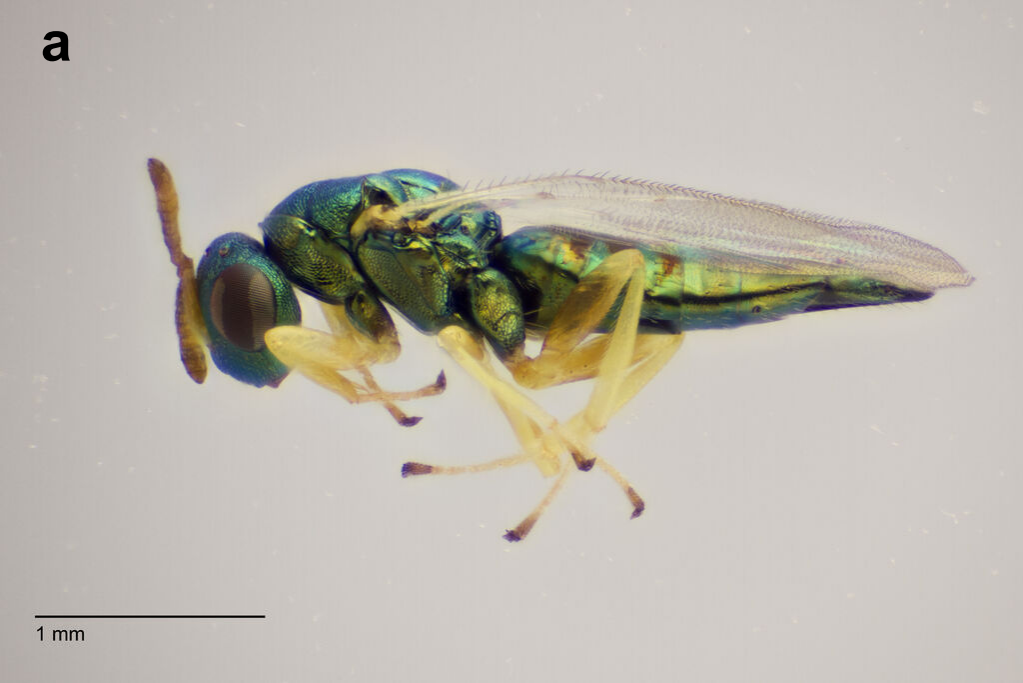
Adult female *Mesopolobusrhabdophagae* (Pteromalidae subfamily Pteromalinae), an idiobiont ectoparasitoid of Rabdophagarosaria, inducing rosette galls of *Salix* sp. Soldaterskov, Denmark.

**Figure 6b.**
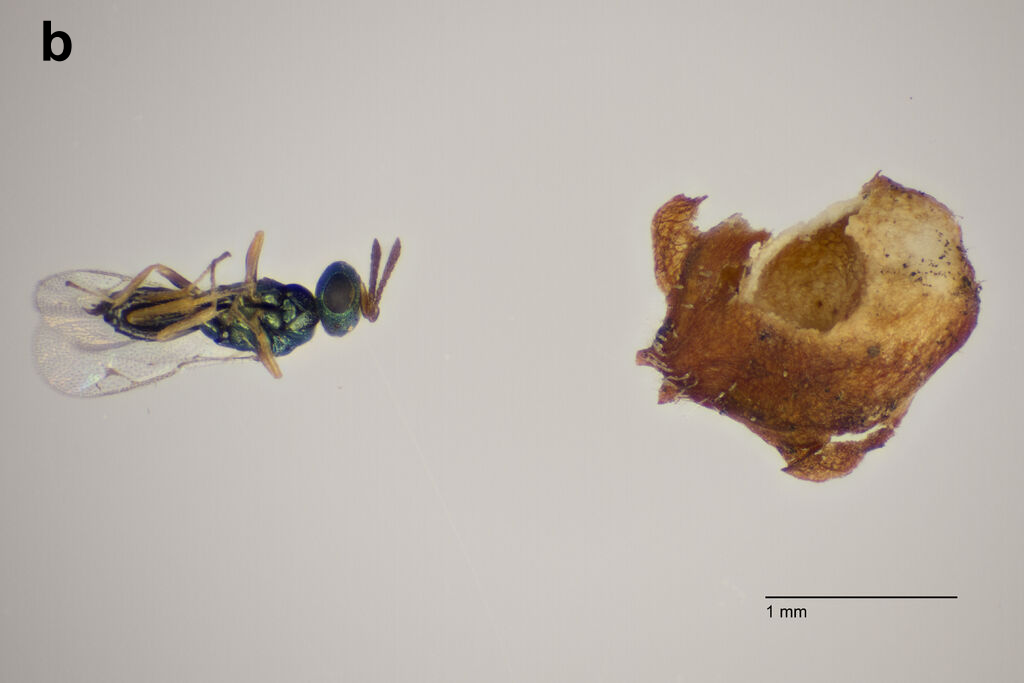
Adult female *Psilonotusachaeus* (Pteromalidae subfamily Pteromalinae), emerged directly from *Semudobiabetulae* induced seed gall in *Betulapendula*. Vingsted, Denmark.

**Figure 6c.**
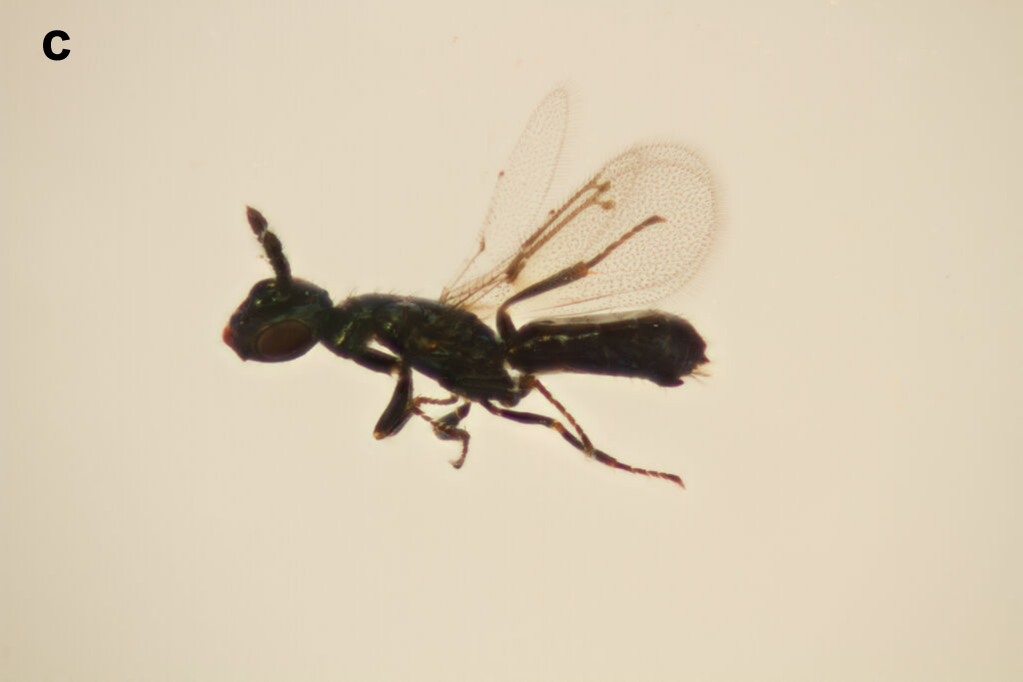
Adult female *Macrogleneschalybeus* (Pteromalidae subfamily Pireninae, syn. Pirenidae) extracted directly from a gall of a *Contariniasteini* flower bud gall on *Silenelatifolia*. Svanninge Bjerge, Denmark.

**Figure 6d.**
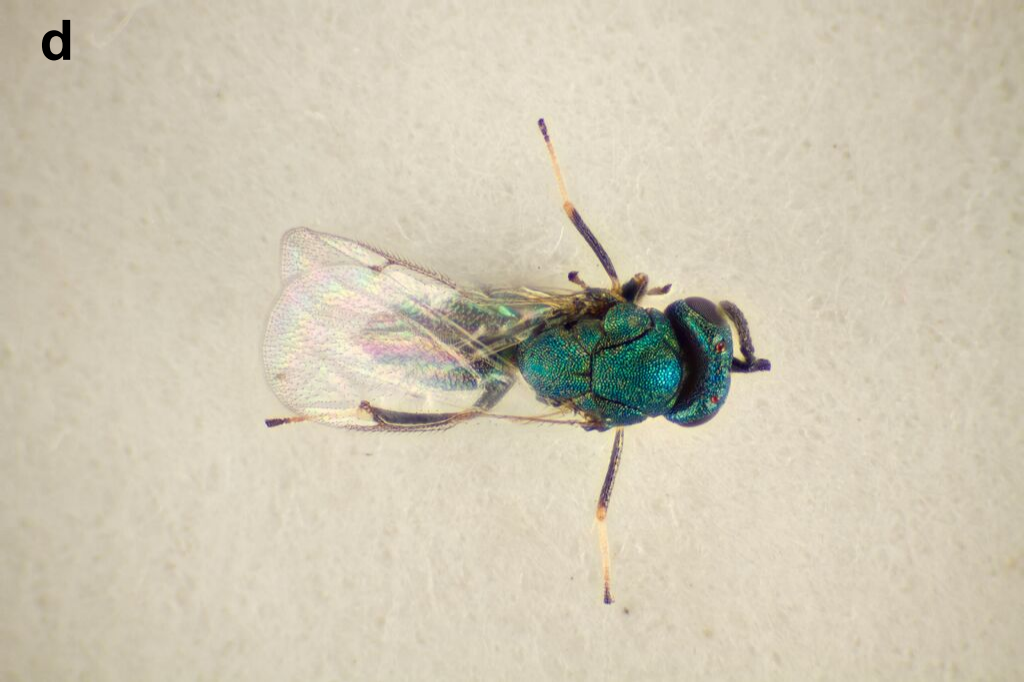
Dorsal view of adult female *Systasisencyrtoides* (Pteromalidae subfamily Systasinae, syn. Systasidae), an idiobiont ectoparasitoid of *Dasineuracrataegi* galling *Crataegus* sp. Vordingborg, Denmark.

**Figure 7a.**
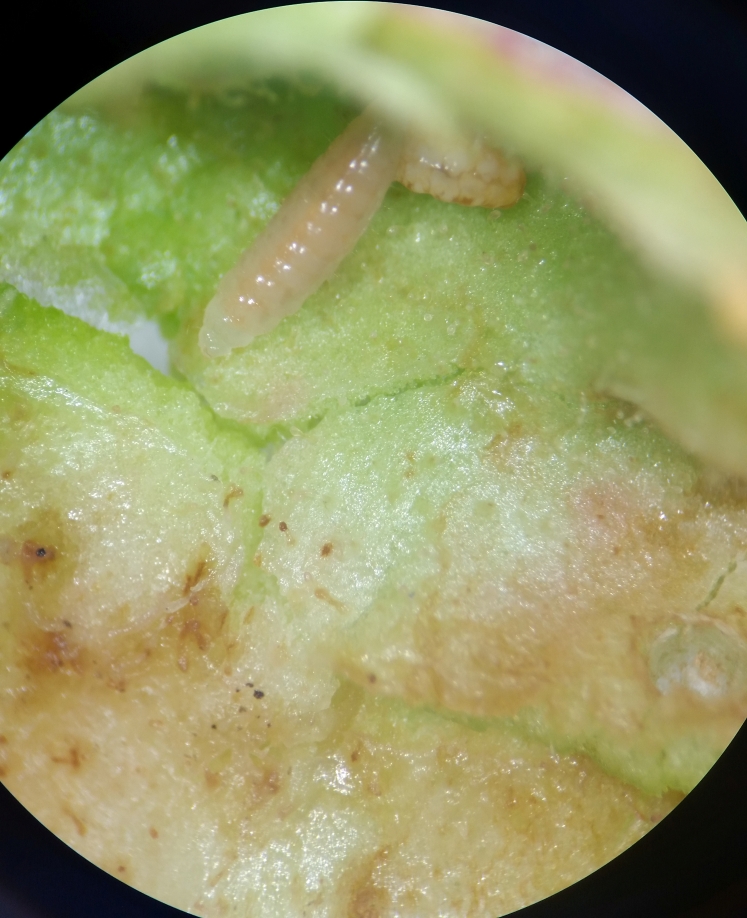
Larva of *Torymuspersicariae* (Torymidae subfamily Toryminae) together with host larvae of *Wachtliellapersicariae* in leaf roll gall on *Persicariaamphibia*. Slangerup, Denmark.

**Figure 7b.**
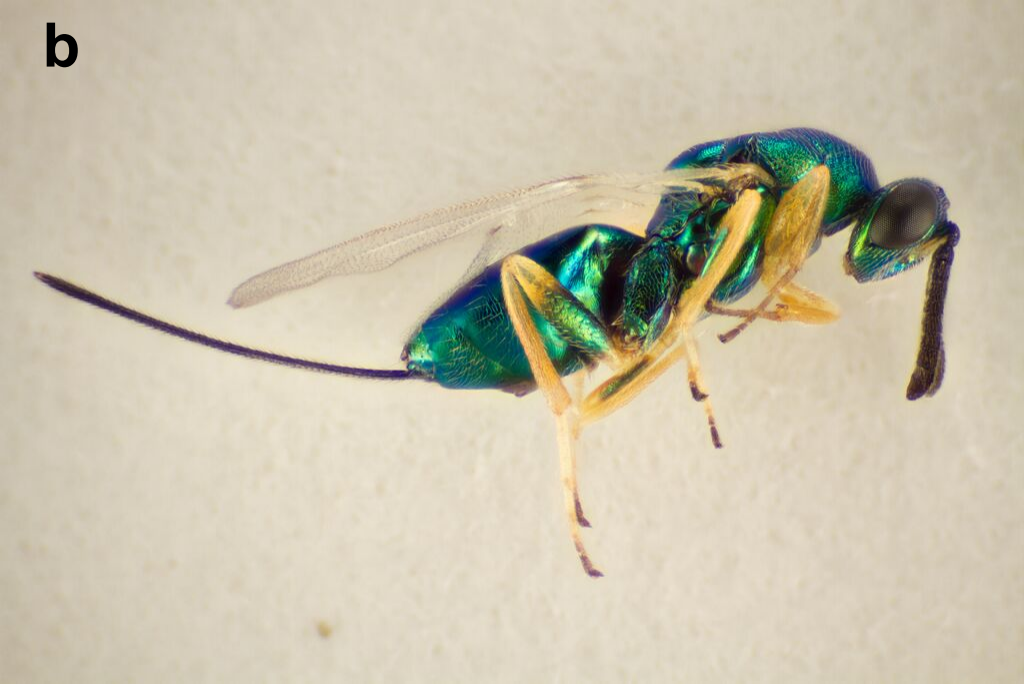
Adult female of the same collection, reared using method 3. Slangerup, Denmark.

**Figure 7c.**
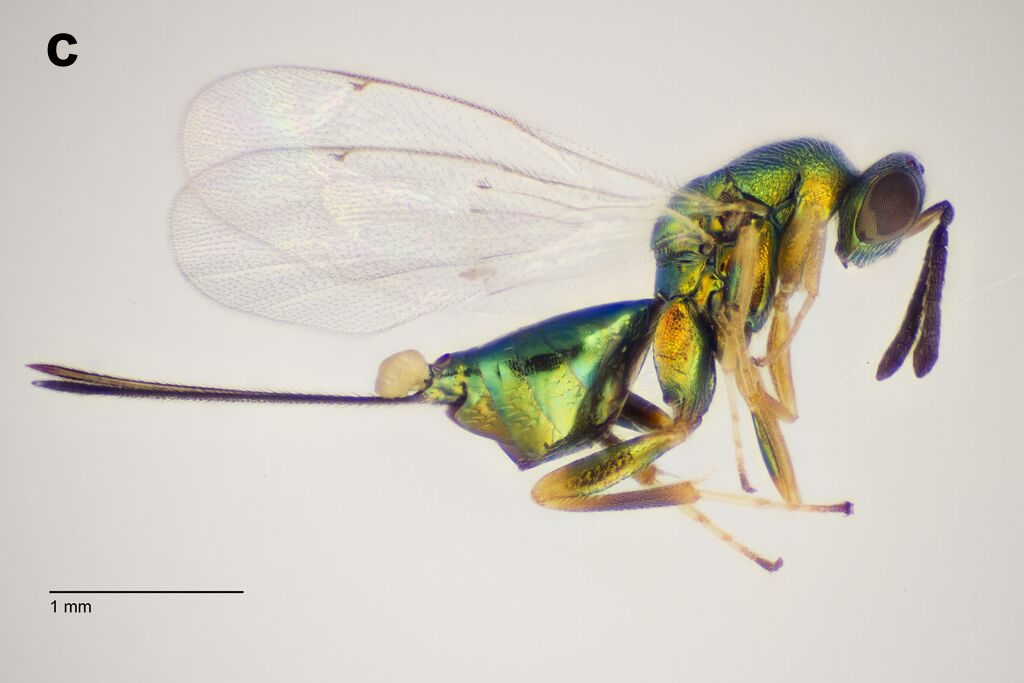
*Torymusanthobiae*, ectoparasitoid of *Contariniaanthobia* gallling *Crataegusmonogyna*. Landbohøjskolen, Denmark.

**Figure 7d.**
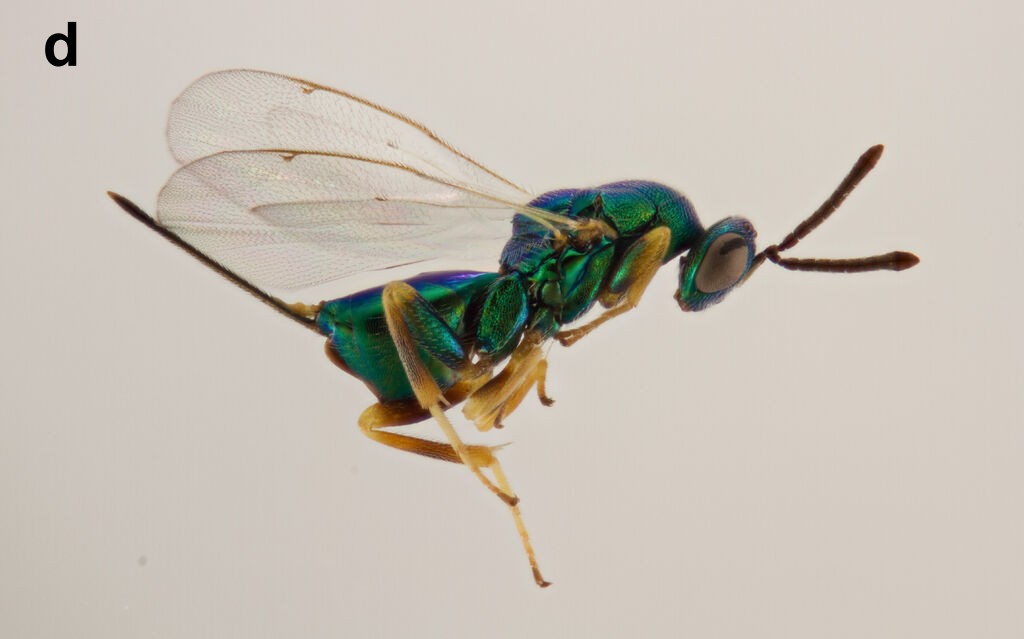
*Torymusrosariae*, ectoparasitoid of *Rabdophagarosaria* galling *Salix* sp. Rask Skov, Denmark.

**Table 1.** Records of hymenopteran parasitoids reared from European Cecidomyiidae. All Ceraphronidae and Platygastridae were identified by Peter Neerup Buhl. With few exceptions, Richard R. Askew identified all Encyrtidae, Eulophidae, Eupelmidae, Eurytomidae, Pteromalidae and Torymidae. Identities of collectors: AM Asta Malakauskienė, AP Alexandru Pintilioaie, BA Belinda Andersen, BK Birgit Knudsen, BWP Brian Willum Petersen, EF Emil Førby, HHB Hans Henrik Bruun, JS Johan Svedholm, KA Ken Alminde, KH Kresten Hansen, KN Klavs Nielsen, LKT Linda Kjær-Thomsen, MB Martin Bjerg, ND Netta Dorchin, PB Peder Brøgger, PBJ Peter Bonde Jensen, SH Simon Haarder. Method of rearing (details in the main text): 1, Adults extracted from galls (1A) or collected while ovipositing (1B); 2, Adults emerged directly from galls; 3, Adults emerged in gelatine capsules; 4, Adults emerged from soil, to which gall midge larvae had been transferred, earlier the same year (4A) or in the preceding year (4B); 5, No rearing of adults. “Emg. month”: Start of emergence (month, year). “C.”: Country. Novelty of interaction (“Int. nov.”): N, New host-parasitoid interaction; FH, First host record for parasitoid species; FCH, First Cecidomyiidae host record for parasitoid species; FP, First parasitoid record for host species. Biogeographic novelty (“Biogeo. nov.”): First record of species for the country indicated. Details of the records (collection site name and geographic coordinates, as well as number of females and males obtained and identity of the gall midge host plant) are given in Suppl. material 1.

**Parasitoid name**	**Gall midge host name**	**Method**	**Collection date**	**Emg. month**	**Coll.**	**C.**	**Int. nov.**	**Biogeo. nov.**
**Ceraphronidae**								
*Aphanogmusabdominalis* (Thomson, 1858)	*Dasineuraodoratae* Stelter, 1982	2	02.ix.2010	NA	HHB	DK	-	-
*Aphanogmusabdominalis* (Thomson, 1858)	*Dasineuraodoratae* Stelter, 1982	2	03.ix.2010	NA	HHB	DK	-	-
*Aphanogmusabdominalis* (Thomson, 1858)	*Dasineuraodoratae* Stelter, 1982	2	19.ix.2010	NA	HHB	DK	-	-
*Aphanogmusabdominalis* (Thomson, 1858)	*Dasineuraodoratae* Stelter, 1982	3	26.xii.2018	i.2019	SH	DK	-	-
*Aphanogmusabdominalis* (Thomson, 1858)	Dasineura violahirtae Stelter, 1982	2	31.iii.2020	iv.2020	HHB	DK	FP	-
*Aphanogmusfasciipennis* Thomson, 1858	Mycodiplosiscf.melampsorae (Rübsaamen, 1889)	4A	04.x.2020	x.2020	HHB	DK	FH	-
Aphanogmuscf.fasciipennis Thomson, 1858	*Contariniafestucae* Jones, 1940	4B	08.vii.2021	v.2022	HHB	DK	N	-
Aphanogmuscf.fasciipennis Thomson, 1858	*Stenodiplosis* sp. ex *Elymusrepens*	4B	18.vii.2021	v.2022	HHB	DK	N	-
*Aphanogmusgracilicornis* Förster, 1861	*Mycodiplosismelampsorae* (Rübsaamen, 1889)	4A	04.x.2020	x.2020	HHB	DK	FH	-
*Aphanogmusmicroneurus* Kieffer, 1907	*Mycodiplosismelampsorae* (Rübsaamen, 1889)	4A	04.x.2020	x.2020	HHB	DK	FH	-
*Aphanogmusvicinus* Förster, 1861	*Mycodiplosismelampsorae* (Rübsaamen, 1889)	4A	04.x.2020	x.2020	HHB	DK	FH	-
**Encyrtidae**								
*Pseudencyrtusmisellus* (Dalman, 1820)	*Rabdophagasalicis* (Schrank, 1803)	NA	03.iv.2021	iv.2021	PBJ	DK	-	-
*Pseudencyrtusmisellus* (Dalman, 1820)	*Rabdophagasalicis* (Schrank, 1803)	3	08.iii.2021	iii.2021	SH	DK	-	-
*Pseudencyrtusmisellus* (Dalman, 1820)	*Rabdophagasalicis* (Schrank, 1803)	NA	14.iv.2020	NA	KH	DK	-	-
**Eulophidae**								
*Aprostocetusaethiops* (Zetterstedt, 1838)	*Contariniapulchripes* (Kieffer, 1890)	3	26.vii.2015	viii.2015	SH	DK	-	DK
*Aprostocetusamenon* (Walker, 1839)	*Dasineuraulmaria* (Bremi, 1847)	NA	14.vii.2021	NA	AP	RO	-	RO
*Aprostocetusanodaphus* (Walker, 1839)	*Ozirhincusmillefolii* (Wachtl, 1884)	2	16.viii.2020	viii.2020	HHB	DK	-	-
*Aprostocetusanodaphus* (Walker, 1839)	*Ozirhincusmillefolii* (Wachtl, 1884)	2	16.viii.2020	viii.2020	HHB	DK	N	DK
*Aprostocetusapama* (Walker, 1839)	*Planetellagranifex* (Kieffer, 1898)	2	06.iv.2016	vi.2016	HHB	DK	FH	DK
*Aprostocetusapama* (Walker, 1839)	*Planetellagallarum* (Rübsaamen, 1899)	2	07.vi.2016	vii.2016	HHB	DK	N	-
*Aprostocetusartemisiae* (Erdös, 1954)	*Rhopalomyiaartemisiae* (Bouché, 1834)	2	08.viii.2022	viii.2022	HHB	DK	N	-
*Aprostocetusartemisiae* (Erdös, 1954)	*Dasineuraartemisiae* (Rübsaamen, 1915)	2	17.viii.2021	viii.2021	HHB	DK	FP	DK
*Aprostocetuscatius* (Walker, 1839)*^2^	*Contariniafestucae* Jones, 1940	4B	08.vii.2021	v.2022	HHB	DK	FHFP	DK
*Aprostocetuscecidomyiarum* (Bouché, 1834)	*Dasineuraartemisiae* (Rübsaamen, 1915)	2	17.viii.2021	viii.2021	HHB	DK	N	DK
*Aprostocetusclavicornis* (Zetterstedt, 1838)	*Semudobiabetulae* (Winnertz, 1853)	2	03.xii.2015	i.2016	SH	DK	-	DK
*Aprostocetusclavicornis* (Zetterstedt, 1838)	*Semudobiabetulae* (Winnertz, 1853)	2	25.xii.2015	i.2016	KA	DK	-	-
*Aprostocetuscrino* (Walker, 1838)	Unknown cecidomyid inquiline ex *Aceriagaliobia*	2	18.vii.2021	vii.2021	HHB	DK	N	-
*Aprostocetusdotus* (Walker, 1839)	*Dasineuraulmaria* (Bremi, 1847)	2	07.vii.2022	vii.2022	HHB	DK	-	DK
*Aprostocetusdotus* (Walker, 1839)	*Dasineuraulmaria* (Bremi, 1847)	2	14.vii.2022	vii.2022	HHB	DK	-	-
*Aprostocetuselongatus* (Förster, 1841)	*Mikiolafagi* (Hartig, 1839)	3	13.ii.2020	iii.2020	SH	DK	-	-
*Aprostocetuselongatus* (Förster, 1841)	*Mikiolafagi* (Hartig, 1839)	3	20.xi.2015	xii.2015	SH	DK	-	-
*Aprostocetuselongatus* (Förster, 1841)	*Mikiolafagi* (Hartig, 1839)	3	22.xi.2017	xii.2017	SH	DK	-	-
*Aprostocetuselongatus* (Förster, 1841)	*Mikiolafagi* (Hartig, 1839)	3	29.i.2015	ii.2015	SH	DK	-	-
*Aprostocetusepicharmus* (Walker, 1839)	*Dasineuraserotina* (Winnertz, 1853)	3	20.vi.2016	vii.2026	SH	DK	FP	DK
*Aprostocetusescherichi* (Szelenyi, 1941)	*Semudobiatarda* Roskam, 1977	3	12.iii.2019	iii.2019	SH	PL	-	PL
*Aprostocetusgratus* (Giraud, 1863)	*Giraudiellainclusa* (Frauenfeld, 1862)	2	12.iii.2021	iv.2021	HHB	DK	-	-
*Aprostocetusluteus* (Ratzeburg, 1848)	*Mikiolafagi* (Hartig, 1839)	3	01.xii.2015	x.2016	SH	DK	-	DK
*Aprostocetuslycidas* (Walker, 1839)	*Hartigiolaannulipes* (Hartig, 1839)	3	01.xi.2015	xii.2015	SH	DK	-	-
*Aprostocetuslycidas* (Walker, 1839)	*Hartigiolaannulipes* (Hartig, 1839)	3	13.x.2020	xi.2021	SH	DK	-	-
*Aprostocetuslysippe* (Walker, 1839)	*Dasineuracrataegi* (Winnertz, 1853)	4B	08.vii.2021	vii.2021	HHB	DK	-	-
*Aprostocetuslysippe* (Walker, 1839)	*Dasineuracrataegi* (Winnertz, 1853)	3	08.viii.2017	viii.2017	SH	DK	-	DK
*Aprostocetusmenius* (Walker, 1839)	*Lasiopteracalamagrostidis* Rübsaamen, 1893	3	27.ii.2015	iv.2015	SH	DK	FH	DK
*Aprostocetusmicantulus* (Thomson, 1878)	*Piceacecisabietiperda* (Henschel, 1880)	3	13.ii.2016	iv.2016	SH	DK	N	DK
*Aprostocetusmicroscopicus* (Rondani, 1877)	*Cystiphorataraxaci* (Kieffer, 1888)	3	08.viii.2018	viii.2028	SH	DK	-	DK
Aprostocetuscf.myrsus (Walker, 1839)	*Contariniarumicis* (Loew, 1850)	3	10.viii.2022	viii.2022	SH	DK	N	DK
*Aprostocetusorithyia* (Walker, 1839)	*Giraudiellainclusa* (Frauenfeld, 1862)	2	10.iii.2019	iii.2019	HHB	DK	-	-
*Aprostocetusorithyia* (Walker, 1839)	*Giraudiellainclusa* (Frauenfeld, 1862)	3	27.i.2016	ii.2016	SH	DK	-	-
*Aprostocetuspallipes* (Dalman, 1820)	*Semudobiaskuhravae* Roskam, 1977	2	03.xii.2015	i.2016	SH	DK	-	DK
*Aprostocetuspallipes* (Dalman, 1820)	*Semudobia* sp. (*betulae* / *tarda*)	2	13.iii.2020	iii.2020	SH	DK	-	-
Aprostocetussp. nrpallipes (Dalman, 1820)	*Iteomyiamajor* (Kieffer, 1889)	3	20.x.2020	xi.2020	SH	DK	N	-
Aprostocetuscf.phineus (Walker, 1839)	*Stenodiplosis* sp. ex *Elymusrepens*	4B	18.vii.2021	iv.2022	HHB	DK	FH	-
*Aprostocetusplaniusculus* (Thomson, 1878)	Planetellacf.tarda (Rübsaamen, 1899)	2	01.v.2019	v.2019	HHB	DK	N	-
*Aprostocetusplaniusculus* (Thomson, 1878)	Planetellacf.gallarum (Rübsaamen, 1899)	2	16.iii.2019	v.2019	HHB	DK	N	-
*Aprostocetusplaniusculus* (Thomson, 1878)	*Planetellagranifex* (Kieffer, 1898)	2	16.iii.2019	v.2019	HHB	DK	-	DK
*Aprostocetusplaniusculus* (Thomson, 1878)	*Planetellagranifex* (Kieffer, 1898)	2	30.iv.2022	v.2022	HHB	DK	-	-
*Aprostocetusrhacius* (Walker, 1839)	*Dasineuraspadicea* Rübsaamen, 1917	2	05.viii.2022	viii.2022	HHB	DK	N	DK
*Aprostocetusrubi* Graham, 1987	*Lasiopterarubi* (Schrank, 1803)	2	01.iv.2020	v.2020	HHB	DK	-	-
*Aprostocetusrubi* Graham, 1987	*Lasiopterarubi* (Schrank, 1803)	3	24.ix.2018	x.2018	SH	DK	-	DK
*Aprostocetusrubicola* Graham, 1987	*Lasiopterarubi* (Schrank, 1803)	3	17.xii.2015	i.2016	KA	DK	-	DK
*Aprostocetusrubicola* Graham, 1987	*Lasiopterarubi* (Schrank, 1803)	3	18.xii.2015	i.2016	KA	DK	-	-
*Aprostocetusrubicola* Graham, 1987	*Lasiopterarubi* (Schrank, 1803)	3	30.iii.2021	iv.2021	SH	DK	-	-
Aprostocetuscf.suevius (Walker, 1839)	*Dasineuraulmaria* (Bremi, 1847)	2	04.vii.2022	vii.2022	HHB	DK	FCH	DK
Aprostocetuscf.suevius (Walker, 1839)	*Dasineuraulmaria* (Bremi, 1847)	2	14.vii.2022	vii.2022	HHB	DK	-	-
*Aprostocetustanaceticola* Graham, 1987	*Rhopalomyiatanaceticola* (Karsch, 1879)	3	18.ix.2016	xii.2016	SH	DK	-	DK
*Aprostocetustanaceticola* Graham, 1987	*Rhopalomyiatanaceticola* (Karsch, 1879)	3	22.viii.2017	ix.2017	KN	DK	-	-
*Aprostocetustanaceticola* Graham, 1987	*Rhopalomyiatanaceticola* (Karsch, 1879)	3	24.ix.2016	x.2016	SH	DK	-	-
*Aprostocetusveronicae* Graham, 1987	*Jaapiellaveronicae* (Vallot, 1827)	3	15.viii.2017	ix.2017	SH	DK	-	DK
*Aprostocetusviridinitens* Graham, 1987	*Anthodiplosisrudimentalis* (Kieffer, 1901)	2	29.viii.2022	ix.2022	HHB	DK	FHFP	DK
*Aprostocetus* sp. A (probably undescribed)	*Asphondyliamenthae* Kieffer, 1902	3	17.xii.2020	i.2021	SH	DK	-	-
*Aprostocetus* sp. B	*Contariniaarrhenatheri* Kieffer, 1901	4B	25.vi.2020	v.2021	HHB	DK	-	-
*Aprostocetus* sp. C	*Contariniaarrhenatheri* Kieffer, 1901	4B	25.vi.2020	v.2021	HHB	DK	-	-
*Aprostocetus* sp. D	*Dasineurafraxini* (Bremi, 1847)	3	08.x.2018	x.2018	SH	DK	-	-
*Aprostocetus* sp. E	*Dasineuraulmaria* (Bremi, 1847)	3	06.viii.2017	viii.2027	SH	DK	-	-
*Aprostocetus* sp. F	*Parallelodiplosisgalliperda* (F. Löw, 1889)	3	25.ix.2018	x.2018	SH	DK	-	-
*Aprostocetus* sp. G	*Rhopalomyiaartemisiae* (Bouché, 1834)	2	14.x.2016	i.2016	MB	DK	-	-
*Aprostocetus* sp. H	*Rhopalomyiaartemisiae* (Bouché, 1834)	2	22.xii.2016	i.2016	MB	DK	-	-
*Aprostocetus* sp. I	*Stenodiplosis* sp. ex *Elymusrepens*	4B	18.vii.2021	v.2022	HHB	DK	-	-
*Aprostocetus* sp. K	*Wachtliellapersicariae* (Linnaeus, 1767)	2	09.viii.2022	viii.2022	HHB	DK	-	-
*Aprostocetus* sp. L	*Contariniaacrocecis* Stelter, 1962	4B	08.vii.2021	iv.2022	HHB	DK	-	-
*Aprostocetus* sp. M	*Contarinia* sp. "*euonymi*" nom.inedit.	4B	13.vi.2021	iii.2022	HHB	DK	-	-
*Aprostocetus* sp. N	*Dasineura* sp. A sensu Harris (2010)	2	20.vi.2020	vii.2020	HHB	DK	-	-
*Aprostocetus* sp. P	*Rhopalomyiatubifex* (Bouché, 1847)	2	06.viii.2020	viii.2020	HHB	DK	-	-
*Asecodescongruens* (Nees, 1834)	*Mikiolafagi* (Hartig, 1839)	3	29.i.2015	ii.2015	SH	DK	FH	
*Euderusalbitarsis* (Zetterstedt, 1838)	*Rabdophagarosaria* (Loew, 1850)	NA	01.iv.2020	iv.2020	KH	DK	N	-
*Ionymphacarne* (Walker, 1839)	Mycodiplosiscf.melampsorae (Rübsaamen, 1889)	4A	04.x.2020	x.2020	HHB	DK	FHFP	DK
*Omphaleaethiops* Graham, 1963	*Dasineuraepilobii* (F. Löw, 1889)	4B	05.vii.2022	iv.2023	HHB	DK	-	-
*Omphaleaethiops* Graham, 1963	*Dasineuraacrophila* (Winnertz, 1853) and *Macrolabispavida* (Winnertz, 1853)	4B	06.vi.2022	iii.2023	HHB	DK	N	
*Omphaleaetius* (Walker, 1839)	*Dasineuratiliae* (Schrank, 1803)	4B	01.vi.2021	iii.2022	HHB	DK	N	-
*Omphaleaetius* (Walker, 1839)	*Dasineuraurticae* (Perris, 1840)	4A	09.20.2020	vii.2020	HHB	DK	-	DK
Omphalecf.aetius (Walker, 1839)	*Dasineuratiliae* (Schrank, 1803)	4B	09.vi.2022	iii.2023	HHB	DK	N	DK
*Omphale* sp. (*aetius* group)	*Rondaniolabursaria* (Bremi, 1847)	4A	11.vii.2020	ix.2020	HHB	DK	-	-
Omphalesp. nrchryseis Graham, 1963	*Diodauluslinariae* (Winnertz, 1853)	4B	09.viii.2022	iii.2023	HHB	DK	N	-
*Omphaleclymene* (Walker, 1839)	*Dasineuraplicatrix* (Loew, 1850)	4B	14.viii.2022	iv.2023	HHB	DK	N	-
Omphalecf.clymene (Walker, 1839)	*Dasineurafructum* (Rübsaamen, 1895)	4B	12.vii.2020	v.2021	HHB	DK	N	-
*Omphaleconnectens* Graham, 1963	*Dasineuraurticae* (Perris, 1840)	4A	09.20.2020	vii.2020	HHB	DK	FH	-
Omphalesp. nrgrahami Gijswijt, 1976	*Monarthropalpusflavus* (Schrank, 1776)	2	13.iii.2022	iii.2022	HHB	DK	N	DK
*Omphalelugens* (Nees, 1834)	*Dasineuratortilis* (Bremi, 1847)	4B	06.vi.2022	iii.2023	HHB	DK	-	-
*Omphalelugens* (Nees, 1834)	*Mikiolafagi* (Hartig, 1839)	3	22.xi.2017	iv.2018	SH	DK	-	-
*Omphalephaola* (Walker, 1839)	*Dasineuraoxyacanthae* Rübsaamen, 1914	4B	05.vi.2022	iii.2023	HHB	DK	FHFP	DK
*Omphalephaola* (Walker, 1839)	*Dasineuratortilis* (Bremi, 1847)	4B	06.vi.2022	iii.2023	HHB	DK	FH	-
*Omphalephruron* (Walker, 1839)	*Dasineuragaliicola* (F. Löw, 1880)	4B	06.vii.2022	iii.2023	HHB	DK	FH	-
*Omphaletelephe* (Walker, 1839)	*Dasineurasaxifragae* (Kieffer, 1891)	4B	01.vi.2020	iii.2021	HHB	DK	FHFP	DK
*Omphaletheana* (Walker, 1839)	*Dasineuraepilobii* (F. Löw, 1889)	4B	05.vii.2022	iv.2023	HHB	DK	N	-
*Omphaletheana* (Walker, 1839)	*Contariniascrophulariae* Kieffer, 1896	4B	08.vii.2022	iii.2023	HHB	DK	-	-
*Omphaletheana* (Walker, 1839)	*Contariniascrophulariae* Kieffer, 1896	1B	19.vi.2021	vi.2021	HHB	DK	FH	DK
*Omphaletheana* (Walker, 1839)	*Contarinia* sp. "*glycyphylli*" nom.inedit.	4B	21.vi.2022	iv.2023	HHB, ND	DK	N	-
*Quadrastichusanysis* (Walker, 1839)	*Monarthropalpusflavus* (Schrank, 1776)	2	12.xii.2014	xii.2014	SH	DK	-	-
*Quadrastichusanysis* (Walker, 1839)	*Monarthropalpusflavus* (Schrank, 1776)	3	12.xii.2014	xii.2014	SH	DK	-	DK
*Quadrastichusanysis* (Walker, 1839)	*Monarthropalpusflavus* (Schrank, 1776)	2	15.iii.2020	iv.2020	HHB	DK	-	-
*Quadrastichusanysis* (Walker, 1839)	*Monarthropalpusflavus* (Schrank, 1776)	3	24.iii.2017	iv.2017	SH	DK	-	-
*Quadrastichuslasiocerus* (Graham, 1961)	*Wachtliellapersicariae* (Linnaeus, 1767)	2	14.vii.2022	vii.2022	HHB	DK	-	DK
*Sigmophorabrevicornis* (Panzer, 1804)	*Asphondyliabaudysi* Vimmer, 1937	3	01.ix.2020	ix.2020	KA	DK	-	-
*Sigmophorabrevicornis* (Panzer, 1804)	*Asphondyliasarothamni* (Loew, 1850)	5	01.vii.2016	NA	SH	DK	-	-
*Sigmophorabrevicornis* (Panzer, 1804)	*Asphondyliasarothamni* (Loew, 1850)	3	02.vii.2016	vii.2016	SH	DK	-	-
*Sigmophorabrevicornis* (Panzer, 1804)	*Asphondyliasarothamni* (Loew, 1850)	5	03.vii.2013	NA	SH	DK	-	-
*Sigmophorabrevicornis* (Panzer, 1804)	*Asphondyliasarothamni* (Loew, 1850)	5	03.vii.2016	NA	SH	DK	-	-
*Sigmophorabrevicornis* (Panzer, 1804)	*Asphondyliasarothamni* (Loew, 1850)	5	03.viii.2020	NA	SH	DK	-	-
*Sigmophorabrevicornis* (Panzer, 1804)	*Asphondyliasarothamni* (Loew, 1850)	5	07.viii.2020	NA	SH	DK	-	-
*Sigmophorabrevicornis* (Panzer, 1804)	*Kiefferiapericarpiicola* (Bremi, 1847)	5	08.viii.2022	NA	SH	DK	-	-
*Sigmophorabrevicornis* (Panzer, 1804)	*Asphondyliasarothamni* (Loew, 1850)	5	12.vi.2017	NA	SH	DK	-	-
*Sigmophorabrevicornis* (Panzer, 1804)	*Asphondyliasarothamni* (Loew, 1850)	5	13.vi.2020	NA	SH	DK	-	-
*Sigmophorabrevicornis* (Panzer, 1804)	*Asphondyliamelanopus* Kieffer, 1890	5	16.viii.2017	NA	SH	DK	-	-
*Sigmophorabrevicornis* (Panzer, 1804)	*Asphondyliasarothamni* (Loew, 1850)	5	17.vii.2016	vii.2016	BA	DK	-	-
*Sigmophorabrevicornis* (Panzer, 1804)	*Asphondyliamenthae* Kieffer, 1902	3	17.xii.2020	i.2021	SH	DK	N	-
*Sigmophorabrevicornis* (Panzer, 1804)	*Asphondyliasarothamni* (Loew, 1850)	3	19.v.2016	v.2016	SH	DK	-	-
*Sigmophorabrevicornis* (Panzer, 1804)	*Kiefferiapericarpiicola* (Bremi, 1847)	2	23.viii.2018	viii.2018	HHB	PL	-	-
*Sigmophorabrevicornis* (Panzer, 1804)	*Kiefferiapericarpiicola* (Bremi, 1847)	1A	24.ix.2016	NA	SH	DK	-	-
*Sigmophorabrevicornis* (Panzer, 1804)	*Asphondyliasarothamni* (Loew, 1850)	5	26.vii.2017	NA	SH	DK	-	-
*Sigmophorabrevicornis* (Panzer, 1804)	*Asphondyliasarothamni* (Loew, 1850)	5	28.vii.2023	NA	SH	DK	-	-
*Sigmophorabrevicornis* (Panzer, 1804)	*Asphondylia* sp.	3	30.ix.2020	x.2020	SH	DK	-	-
*Sigmophorabrevicornis* (Panzer, 1804)	*Asphondyliasarothamni* (Loew, 1850)	3	30.vi.2016	vii.2016	SH	DK	-	-
**Eupelmidae**								
Eupelmuscf.tremulae Delvare 2014*^7^	*Harmandiolacavernosa* (Rübsaamen, 1899)	1A	10.viii.2018	viii.2018	HHB	DK	FP	DK
*Eupelmusconfusus* Al khatib, 2015	*Asphondyliasarothamni* (Loew, 1850)	3	22.vii.2017	viii.2017	SH	DK	N	-
*Eupelmusconfusus* Al khatib, 2015	*Rhopalomyiaartemisiae* (Bouché, 1834)	2	22.xii.2016	i.2016	MB	DK	N	DK
*Eupelmusconfusus* Al khatib, 2015	*Asphondyliasarothamni* (Loew, 1850)	3	26.vii.2017	viii.2017	SH	DK	-	-
*Eupelmusurozonus* Dalman, 1820	*Lasiopterarubi* (Schrank, 1803)	3	30.iii.2021	iv.2021	SH	DK	-	-
*Eupelmusurozonus* sensu lato	*Mikiolafagi* (Hartig, 1839)	3	20.xi.2015	xii.2015	SH	DK	-	-
*Eupelmusvesicularis* (Retzius, 1783)	*Lasiopterarubi* (Schrank, 1803)	3	30.iii.2021	iv.2021	SH	DK	-	-
**Eurytomidae**								
*Eurytomadentata* Mayr, 1878	*Asphondyliasarothamni* (Loew, 1850)	1A	04.viii.2020	NA	SH	DK	-	-
*Eurytomadentata* Mayr, 1878	*Asphondyliasarothamni* (Loew, 1850)	1A	07.viii.2020	NA	SH	DK	-	-
*Eurytomadentata* Mayr, 1878	*Asphondyliasarothamni* (Loew, 1850)	3	21.xii.2020	i.2021	SH	DK	-	-
*Eurytomadentata* Mayr, 1878	*Asphondyliasarothamni* (Loew, 1850)	3	22.vii.2017	viii.2017	SH	DK	-	DK
*Sycophilafasciata* (Thomson, 1876)	*Giraudiellainclusa* (Frauenfeld, 1862)	2	12.iii.2021	iv.2021	HHB	DK	-	-
**Platygastridae**								
*Acerotella* sp. (*evanescens* group)	*Dasineurapulsatillae* (Kieffer, 1894)	4B	13.vi.2021	iv.2022	HHB	DK	FH	-
*Anopediaslacustris* (Kieffer, 1926)	*Planetellagranifex* (Kieffer, 1898)	2	06.iv.2016	vi.2016	HHB	DK	FH	-
*Anopediaslacustris* (Kieffer, 1926)	*Planetellagranifex* (Kieffer, 1898)	2	16.iii.2019	v.2019	HHB	DK	-	-
*Anopediaslacustris* (Kieffer, 1926)	*Planetellagranifex* (Kieffer, 1898)	2	17.iii.2019	iv.2019	HHB	DK	-	-
*Anopediaslacustris* (Kieffer, 1926)	*Planetellagranifex* (Kieffer, 1898)	2	30.iii.2019	v.2019	HHB	DK	-	-
*Anopediaslacustris* (Kieffer, 1926)	Planetellacf.gallarum (Rübsaamen, 1899)	2	30.iv.2022	v.2022	HHB	DK	N	-
*Anopediasobscurus* Thomson, 1859	*Planetellagallarum* (Rübsaamen, 1899)	2	07.vi.2016	vi.2016	HHB	DK	FP	-
*Anopediasobscurus* Thomson, 1859	*Planetellagranifex* (Kieffer, 1898)	2	30.iii.2019	v.2019	HHB	DK	FH	-
*Anopediassundholmi* Huggert, 1974	*Planetellagranifex* (Kieffer, 1898)	2	30.iii.2019	v.2019	HHB	DK	FH	-
*Inostemmaboscii* (Jurine, 1807) sensu Kozlov 1978	*Dasineuraepilobii* (F. Löw, 1889)	4B	05.vii.2022	iv.2023	HHB	DK	N	-
Inostemmasp. nrboscii (Jurine, 1807) sensu Kozlov 1978	*Contarinia* sp. "*glycyphylli*" nom.inedit.	4B	21.vi.2022	iv.2023	HHB, ND	DK	N	-
*Inostemmakoponeni* Buhl, 2005	*Dasineuramedicaginis* (Bremi, 1847)	4A	10.vii.2020	viii.2020	HHB	DK	FH	DK
*Inostemmalycon* Walker, 1835 sensu Szelényi 1938	*Stenodiplosis* sp. ex *Elymusrepens*	4B	18.vii.2021	v.2022	HHB	DK	FH	DK
*Inostemmawalkeri* Kieffer, 1914 sensu Szelényi 1938	*Dasineurapulsatillae* (Kieffer, 1894)	4B	13.vi.2021	v.2022	HHB	DK	FP	-
*Inostemmawalkeri* Kieffer, 1914 sensu Szelényi 1938	*Contarinia* "*quercuscupuli*" nom.inedit.	2	14.vii.2015	iii.2016	HHB	DK	FP	-
*Inostemmawalkeri* Kieffer, 1914 sensu Szelényi 1938	*Lathyromyzaflorum* Rübsaamen, 1916	4B	15.viii.2022	v.2023	HHB	DK	N	-
Inostemmasp. nrwalkeri Kieffer, 1914	*Dasineurafructum* (Rübsaamen, 1895)	4B	12.vii.2020	v.2021	HHB	DK	N	-
*Isocybusocellaris* Kieffer, 1916	*Planetellaarenariae* (Rübsaamen, 1899)	2	04.x.2020	iv.2021	HHB	DK	FH	-
*Leptacistipulae* (Kirby, 1798)	*Contariniaquinquenotata* (F. Löw, 1888)	2	04.vii.2021	vii.2021	HHB	DK	N	-
*Leptacistipulae* (Kirby, 1798)	*Contariniaquinquenotata* (F. Löw, 1888)	2	05.vii.2021	vii.2021	HHB	DK	-	-
*Leptacistipulae* (Kirby, 1798)	*Contarinia* sp. "*glycyphylli*" nom.inedit.	4B	21.vi.2022	iv.2023	HHB, ND	DK	N	-
*Leptacisvlugi* Buhl, 1997	Mycodiplosiscf.melampsorae (Rübsaamen, 1889)	4A	12.viii.2021	ix.2021	HHB	SE	FH	-
*Metaclisisgermanica* Buhl, 2019	*Dasineuraminungula* Stelter, 1986	2	03.viii.2022	iii.2023	HHB	DK	FHFP	-
*Metaclisismontagnei* Maneval, 1936	*Dasineuraepilobii* (F. Löw, 1889)	4B	05.vii.2022	iv.2023	HHB	DK	FH	DK
*Metaclisisphragmitis* Debauche, 1947	*Semudobiatarda* Roskam, 1977	1A	ultimo.iii.2022	NA	SH	HU	N	HU
*Platygasterathamas* Walker, 1835	*Contarinia* "*quercuscupuli*" nom.inedit.	2	14.vii.2015	iii.2016	HHB	DK	N	-
Platygastersp. nrathamas Walker, 1835	*Dasineuraurticae* (Perris, 1840)	4A	09.20.2020	vii.2020	HHB	DK	N	-
*Platygasterbetulae* (Kieffer, 1916)	*Semudobiabetulae* (Winnertz, 1853)	2	10.iv.2012	iv.2012	HHB	DK	-	-
*Platygasterbetulae* (Kieffer, 1916)	*Semudobiabetulae* (Winnertz, 1853)	1A	14.iii.2014	NA	SH	DK	-	-
*Platygasterbetulae* (Kieffer, 1916)	*Semudobiatarda* Roskam, 1977	1A	medio.ix.2021	NA	AM	LT	N	LT
*Platygastercompressicornis* (Thomson, 1859)	*Planetellaarenariae* (Rübsaamen, 1899)	2	10.v.2020	v.2020	HHB	DK	N	-
*Platygasterdamokles* (Buhl, 1998)	*Dasineuraepilobii* (F. Löw, 1889)	4B	05.vii.2022	iv.2023	HHB	DK	FH	-
*Platygasterdemades* Walker, 1835	*Dasineuraurticae* (Perris, 1840)	4A	06.20.2020	vii.2020	HHB	DK	N	-
*Platygasterdemades* Walker, 1835	*Dasineuraurticae* (Perris, 1840)	4A	09.20.2020	vii.2020	HHB	DK	-	-
*Platygasterdemades* Walker, 1835	*Contarinia* "*quercuscupuli*" nom.inedit.	2	14.vii.2015	iii.2016	HHB	DK	N	-
*Platygasterdemades* Walker, 1835	*Lasiopterarubi* (Schrank, 1803)	3	30.iii.2021	iv.2021	SH	DK	N	-
*Platygasterdryomyiae* Silvestre, 1916	*Dryomyialichtensteinii* (F. Löw, 1878)	2	01.iv.2018	iv.2018	HHB	ES	-	-
*Platygasterdryomyiae* Silvestre, 1916	*Dryomyialichtensteinii* (F. Löw, 1878)	2	02.iv.2018	iv.2018	HHB	ES	-	-
*Platygasterdryope* Walker, 1836	Mycodiplosiscf.melampsorae (Rübsaamen, 1889)	4A	12.viii.2021	ix.2021	HHB	SE	FH	-
*Platygasterentwistlei* Buhl, 1997	*Oligotrophusvalerii* (Tavares, 1904)	2	23.iv.2019	vi.2019	HHB	ES	FHFP	ES
*Platygasterentwistlei* Buhl, 1997	*Oligotrophusvalerii* (Tavares, 1904)	2	26.iv.2019	vi.2019	HHB	ES	-	-
*Platygastereriphyle* Walker, 1835	*Rabdophagasalicis* (Schrank, 1803)	1A	15.i.2018	NA	SH	DK	N	-
*Platygastereuhemerus* Walker, 1835	*Ametrodiplosisthalictricola* (Rübsaamen, 1895)	2	18.vii.2021	v.2022	HHB	DK	FH	-
Platygastersp. nrjutlandica Buhl, 2006	*Putoniellapruni* (Kaltenbach, 1872)	4B	13.vi.2021	iv.2022	HHB	DK	FH	-
*Platygasterleptines* Walker, 1835	*Dasineurasaxifragae* (Kieffer, 1891)	4B	01.vi.2020	iii.2021	HHB	DK	FH	-
*Platygasterleptines* Walker, 1835	*Dasineuraaparines* (Kieffer, 1889)	4B	05.vii.2021	v.2022	HHB	DK	FP	-
*Platygasterleptines* Walker, 1835	*Dasineuraacrophila* (Winnertz, 1853) and *Macrolabispavida* (Winnertz, 1853)	4B	06.vi.2022	iii.2023	HHB	DK	N	-
*Platygastermarginata* Thomson, 1859	*Contarinia* sp. "*uliginosi*" nom. inedit.	4B	27.vi.2019	iv.2020	HHB	DK	FH	-
Platygastersp. nrmunita Walker, 1835	*Dasineurainflata* Stelter, 1986	4B	05.vii.2021	iv.2022	HHB	DK	FP	-
Platygastersp. nrmunita Walker, 1835	*Dasineurainflata* Stelter, 1986	4B	18.vii.2021	iv.2022	HHB	DK	-	-
*Platygasteroebalus* Walker, 1835	*Dasineurafructum* (Rübsaamen, 1895)	4B	04.vii.2022	iii.2023	HHB	DK	N	-
*Platygasteroebalus* Walker, 1835	*Lathyromyzaschlechtendali* (Kieffer, 1886)	4B	18.vi.2022	iii.2023	HHB	SE	FP	-
Platygastersp. nroebalus Walker, 1835	*Dasineurafructicola* (Kieffer, 1909)	2	14.vii.2022	iii.2023	HHB	DK	FP	-
*Platygasteroeclus* Walker, 1835	*Janetiellaglechomae* Tavares, 1930	4B	19.vi.2020	iii.2021	HHB	DK	FHFP	-
Platygastercf.oeclus Walker, 1835*^4^	*Dasineuraglechomae* (Kieffer, 1889)	4B	21.vi.2020	iii.2021	HHB	DK	N	-
*Platygasterpelias* Walker, 1835	*Dasineuraoxyacanthae* Rübsaamen, 1914	4B	05.vi.2022	iii.2023	HHB	DK	N	-
*Platygasterphragmitiphila* Buhl, 2006	*Lasiopteraarundinis* Schiner, 1854	NA	02.iv.2018	iv.2018	LKT	DK	-	-
*Platygasterphragmitiphila* Buhl, 2006	*Lasiopteraarundinis* Schiner, 1854	2	07.v.2017	v.2017	SH	DK	FH	DK
*Platygasterphragmitiphila* Buhl, 2006	*Lasiopteraarundinis* Schiner, 1854	2	10.iii.2019	v.2019	HHB	DK	-	-
*Platygasterphragmitiphila* Buhl, 2006	*Lasiopteraarundinis* Schiner, 1854	2	16.iii.2019	iii.2019	SH	PL	-	PL
*Platygasterrobiniae* Buhl & Duso, 2008	*Obolodiplosisrobiniae* (Haldeman, 1847)	2	04.xi.2019	xii.2019	SH	DK	-	-
*Platygasterrobiniae* Buhl & Duso, 2008	*Obolodiplosisrobiniae* (Haldeman, 1847)	3	09.x.2019	NA	JS	SE	-	-
*Platygasterrobiniae* Buhl & Duso, 2008	*Obolodiplosisrobiniae* (Haldeman, 1847)	3	17.xi.2018	NA	SH	SE	-	SE
*Platygastersagana* Walker, 1835	*Contariniaacrocecis* Stelter, 1962	4B	08.vii.2021	iv.2022	HHB	DK	N	-
*Platygastersagana* Walker, 1835	*Contariniafestucae* Jones, 1940	4B	08.vii.2021	v.2022	HHB	DK	N	-
*Platygastersagana* Walker, 1835	*Dasineurainflata* Stelter, 1986	4B	18.vii.2021	iv.2022	HHB	DK	N	-
Platygastersp. nrsagana Walker, 1835	*Ametrodiplosisthalictricola* (Rübsaamen, 1895)	2	18.vii.2021	v.2022	HHB	DK	N	-
*Platygaster* sp. (*splendidula* group)	*Mayetiolaphalaris* Barnes, 1927	3	02.v.2016	v.2016	SH	DK	-	-
*Platygaster* sp. (*splendidula* group)	*Planetellaarenariae* (Rübsaamen, 1899)	2	04.x.2020	iv.2021	HHB	DK	-	-
*Platygaster* sp. (*splendidula* group)	Mayetiola ventricola (Rübsaamen, 1899)	2	17.iii.2020	iv.2020	HHB	DK	-	-
*Platygasterszelenyii* Huggert, 1975	*Giraudiellainclusa* (Frauenfeld, 1862)	2	10.iii.2019	iii.2019	HHB	DK	-	-
*Platygasterszelenyii* Huggert, 1975	*Giraudiellainclusa* (Frauenfeld, 1862)	2	12.iii.2021	v.2021	HHB	DK	-	-
*Platygasteruniformis* Buhl, 2006	*Rabdophagadubiosa* Kieffer, 1913	2	16.iii.2019	iii.2019	HHB	DK	FH	-
*Platygaster* sp. A	*Dasineurarosae* (Bremi, 1847)	1A	09.viii.2019	NA	SH	DK	-	-
*Platygaster* sp. B	*Dasineuraacrophila* (Winnertz, 1853) and *Macrolabispavida* (Winnertz, 1853)	4B	06.vi.2022	iii.2023	HHB	DK	-	-
*Platygaster* sp. C	*Lathyromyzaflorum* Rübsaamen, 1916	4B	14.vii.2022	iv.2023	HHB	DK	-	-
Platygastridae indet.	*Dasineuragaliicola* (F. Löw, 1880)	4B	06.vii.2022	iii.2023	HHB	DK	-	-
*Synopeasciliatum* Thomson, 1859	Mycodiplosiscf.melampsorae (Rübsaamen, 1889)	4A	12.viii.2021	ix.2021	HHB	SE	FH	-
*Synopeasconvexum* Thomson, 1859	*Dasineurahygrophila* (Mik, 1883)	2	10.viii.2022	viii.2022	HHB	DK	FHFP	DK
*Synopeasgibberosum* Buhl, 1997	*Dasineuraulmaria* (Bremi, 1847)	2	07.vii.2022	vii.2022	HHB	DK	FH	-
*Synopeasgibberosum* Buhl, 1997	*Dasineuraulmaria* (Bremi, 1847)	2	14.vii.2022	vii.2022	HHB	DK	N	-
*Synopeasinerme* Thomson, 1859	*Contariniasolani* (Rübsaamen, 1892)	4A	02.vii.2022	vii.2022	HHB	DK	N	-
*Synopeasinerme* Thomson, 1859	*Dasineuraangelicae* Rübsaamen, 1916	4B	08.viii.2021	v.2022	HHB	SE	FP	-
Synopeassp. nrinerme Thomson, 1859	*Dasineurafructum* (Rübsaamen, 1895)	4B	04.vii.2022	iii.2023	HHB	DK	N	-
*Synopeaslarides* (Walker, 1835)	*Contariniatiliarum* (Kieffer, 1890)	4B	01.vi.2021	iii.2022	HHB	DK	N	-
*Synopeaslarides* (Walker, 1835)	*Dasineurafructum* (Rübsaamen, 1895)	4B	04.vii.2022	iii.2023	HHB	DK	FP	-
*Synopeaslarides* (Walker, 1835)	*Dasineurathomasiana* (Kieffer, 1888)	4B	14.vi.2021	iv.2022	HHB	DK	FP	-
*Synopeasmyles* (Walker, 1835)	*Lathyromyzaflorum* Rübsaamen, 1916	2	14.vii.2022	vii.2022	HHB	DK	N	-
*Synopeasmyles* (Walker, 1835)	*Contariniamedicaginis* Kieffer, 1895	2	14.viii.2020	ix.2020	HHB	DK	N	-
Synopeassp. nrmyles (Walker, 1836)	*Dasineuracrataegi* (Winnertz, 1853)	1A	08.viii.2017	NA	SH	DK	N	-
Synopeassp. nrmyles (Walker, 1836)	*Lathyromyzaflorum* Rübsaamen, 1916	4B	14.vii.2022	iv.2023	HHB	DK	N	-
*Synopeasrhanis* (Walker, 1835)	*Dasineuraurticae* (Perris, 1840)	4A	09.20.2020	vii.2020	HHB	DK	-	-
Synopeassp. (subgenus Sactogaster)*^9^	*Contariniasolani* (Rübsaamen, 1892)	4B	02.vii.2022	iv.2023	HHB	DK	-	-
*Synopeassosis* (Walker, 1835)	*Contariniafagi* Rübsaamen, 1921	1A	07.viii.2020	NA	SH	DK	N	-
*Synopeassosis* (Walker, 1835)	*Wachtliellapersicariae* (Linnaeus, 1767)	2	14.vii.2022	vii.2022	HHB	DK	N	-
*Synopeassosis* (Walker, 1835)	*Contarinia* sp. "*glycyphylli*" nom.inedit.	4B	21.vi.2022	iv.2023	HHB, ND	DK	N	-
*Synopeassubaequalis* (Förster, 1856)	*Contarinia* sp. "*hesperidis*" nom. inedit.	4B	13.vi.2021	iii.2022	EF	DK	FH	-
*Synopeasventrale* (Westwood, 1833)	*Contariniatiliarum* (Kieffer, 1890)	4B	01.vi.2021	iii.2022	HHB	DK	FH	-
**Pteromalidae**								
*Gastrancistrusacontes* Walker, 1840	*Dasineuraacrophila* (Winnertz, 1853) and *Macrolabispavida* (Winnertz, 1853)	4B	06.vi.2022	iii.2023	HHB	DK	FH	DK
*Gastrancistrusaffinis* Graham, 1969*^1^	*Contariniaanthobia* (F. Löw, 1877)	4B	12.vi.2021	iv.2022	HHB	DK	FCH	DK
*Gastrancistrusaffinis* Graham, 1969	*Lathyromyzaflorum* Rübsaamen, 1916	4B	15.viii.2022	iv.2023	HHB	DK	N	-
Gastrancistrussp. nraffinis Graham	*Lathyromyzaflorum* Rübsaamen, 1916	4A	15.viii.2022	ix.2022	HHB	DK	-	DK
*Gastrancistrusglabellus* (Nees, 1834)	*Diodauluslinariae* (Winnertz, 1853)	4B	09.viii.2022	iii.2023	HHB	DK	N	-
*Gastrancistrusglabellus* (Nees, 1834)	*Lathyromyzaflorum* Rübsaamen, 1916	4B	14.vii.2022	iv.2023	HHB	DK	FHFP	DK
*Gastrancistrussalicis* (Nees, 1834)	*Rabdophagasalicis* (Schrank, 1803)	3	13.iii.2015	iv.2015	SH	DK	-	-
*Gastrancistrussalicis* (Nees, 1834)	*Rabdophagadubiosa* Kieffer, 1913	2	16.iii.2019	iii.2019	HHB	DK	N	-
*Gastrancistrussalicis* (Nees, 1834)	*Rabdophagasalicis* (Schrank, 1803)	3	29.ii.2016	NA	LKT	DK	-	-
*Gastrancistrusunicolor* Walker, 1834	*Lathyromyzaschlechtendali* (Kieffer, 1886)	4B	18.vi.2022	iii.2023	HHB	SE	FH	-
*Gastrancistrus* sp. (*vagans* group)	*Dasineuraepilobii* (F. Löw, 1889)	4B	05.vii.2022	vii.2022	HHB	DK	N	-
*Gastrancistrus* sp. (*vagans* group)	*Dasineuraplicatrix* (Loew, 1850)	4B	14.viii.2022	iv.2023	HHB	DK	-	-
*Gastrancistrus* sp. (*vagans* group)	*Contarinia* sp. "*glycyphylli*" nom.inedit.	4B	21.vi.2022	vii.2022	HHB, ND	DK	-	-
Gastrancistrussp. nrvulgaris Walker, 1834	*Dasineurafructicola* (Kieffer, 1909)	2	14.vii.2022	iii.2023	HHB	DK	FH	DK
*Gastrancistrus* sp. (‘sp. indet’ in Graham’s (1969) key)	*Contarinia* sp. "*glycyphylli*" nom.inedit.	4B	21.vi.2022	iv.2023	HHB, ND	DK	-	-
*Gastrancistrus* sp. A	*Dasineuraaparines* (Kieffer, 1889)	4B	05.vii.2021	v.2022	HHB	DK	-	-
*Gastrancistrus* sp. B*^8^	*Planetellaarenariae* (Rübsaamen, 1899)	2	04.x.2020	v.2021	HHB	DK	-	-
*Lampotermabianellatum* Graham, 1969*^5^	*Dasineurainflata* Stelter, 1986	4B	18.vii.2021	v.2022	HHB	DK	-	DK
Lamprotatuscf.pschorni (Delucchi, 1953) sensu Graham 1969	*Contarinia* sp. "*glycyphylli*" nom.inedit.	4B	21.vi.2022	iv.2023	HHB, ND	DK	FH	DK
*Macroglenesbouceki* (Graham, 1969)	*Dasineurarosae* (Bremi, 1847)	1A	03.viii.2020	NA	SH	DK	FH	DK
*Macrogleneschalybeus* (Haliday, 1833)	*Contariniasteini* (Karsch, 1881)	1A	03.viii.2020	NA	SH	DK	FP	DK
*Macrogleneseximius* (Haliday, 1833)	*Contariniaperplicata* Fedotova, 1997	4B	07.vi.2020	vi.2021	HHB	DK	FP	-
*Macrogleneseximius* (Haliday, 1833)	*Contariniaacrocecis* Stelter, 1962	4B	08.vii.2021	iv.2022	HHB	DK	FP	-
*Macroglenespenetrans* (Kirby, 1800)*^3^	*Contarinia* sp. "*holci*" nom.inedit	4B	11.vii.2020	v.2021	HHB	DK	N	DK
*Mesopolobusaspilus* (Walker, 1835)	*Oligotrophusjuniperinus* (Linnaeus, 1758)	3	15.v.2017	v.2017	SH	DK	-	-
*Mesopolobusdiffinis* (Walker, 1834)	*Dasineuratrifolii* (F. Löw, 1874)	3	11.ix.2016	ix.2016	SH	DK	N	-
*Mesopolobusdiffinis* (Walker, 1834)	*Wachtliellapersicariae* (Linnaeus, 1767)	2	14.vii.2022	vii.2022	HHB	DK	N	-
*Mesopolobusdiffinis* (Walker, 1834)	*Rhopalomyiaartemisiae* (Bouché, 1834)	2	14.x.2016	i.2016	MB	DK	N	-
*Mesopolobusdiffinis* (Walker, 1834)	*Rhopalomyiaartemisiae* (Bouché, 1834)	2	17.xii.2016	i.2016	MB	DK	-	-
*Mesopolobusdiffinis* (Walker, 1834)	*Rhopalomyiaartemisiae* (Bouché, 1834)	2	21.xi.2016	i.2016	MB	DK	-	-
*Mesopolobusdiffinis* (Walker, 1834)	*Rhopalomyiatanaceticola* (Karsch, 1879)	3	22.viii.2017	ix.2017	KN	DK	-	-
*Mesopolobusdiffinis* (Walker, 1834)	*Rhopalomyiaartemisiae* (Bouché, 1834)	2	22.xii.2016	i.2016	MB	DK	-	-
*Mesopolobusdiffinis* (Walker, 1834)	*Rhopalomyiaartemisiae* (Bouché, 1834)	2	24.xii.2016	i.2016	MB	DK	-	-
*Mesopolobusdiffinis* (Walker, 1834)	*Rhopalomyiaartemisiae* (Bouché, 1834)	2	25.x.2016	i.2016	MB	DK	-	-
*Mesopolobusfagi* Askew and Lampe, 1998	*Mikiolafagi* (Hartig, 1839)	3	01.i.2017	iii.2017	SH	DK	-	-
*Mesopolobusfagi* Askew and Lampe, 1998	*Mikiolafagi* (Hartig, 1839)	3	03.x.2014	x.2014	SH	DK	-	DK
*Mesopolobusmediterraneus* (Mayr, 1903)	*Oligotrophusvalerii* (Tavares, 1904)	2	26.iv.2019	vi.2019	HHB	ES	N	-
*Mesopolobusnobilis* (Walker, 1834)	*Contariniaarrhenatheri* Kieffer, 1901	4B	25.vi.2020	v.2021	HHB	DK	FCH	DK
*Mesopolobusrhabdophagae* (Graham, 1957)	*Rabdophagarosaria* (Loew, 1850)	3	14.i.2016	i.2016	SH	DK	N	DK
*Mesopolobussemiclavatus* (Ratzeburg, 1848)	*Iteomyiamajor* (Kieffer, 1889)	3	20.x.2020	xi.2020	SH	DK	-	DK
*Mesopolobusteliformis* (Walker, 1834)	*Stenodiplosis* sp. ex *Elymusrepens*	2	18.vii.2021	vii.2021	HHB	DK	FCH	DK
*Mesopolobus* sp. (probably undescribed)	*Dasineuragaliicola* (F. Löw, 1880)	4B	06.vii.2022	iii.2023	HHB	DK	-	-
*Pseudocatolaccusnitescens* (Walker, 1834)	*Asphondyliasarothamni* (Loew, 1850)	1A	07.viii.2020	NA	SH	DK	-	-
*Pseudocatolaccusnitescens* (Walker, 1834)	*Asphondyliasarothamni* (Loew, 1850)	3	26.vii.2017	viii.2017	SH	DK	-	-
*Psilonotusachaeus* Walker, 1848	*Semudobiaskuhravae* Roskam, 1977	2	03.xii.2015	i.2016	SH	DK	-	-
*Psilonotusachaeus* Walker, 1848	*Semudobia* sp. (*betulae* / *tarda*)	2	13.iii.2020	iv.2020	SH	DK	-	-
*Psilonotusachaeus* Walker, 1848	*Semudobiabetulae* (Winnertz, 1853)	2	17.xii.2015	i.2016	KA	DK	-	-
*Psilonotusachaeus* Walker, 1848	*Semudobiatarda* Roskam, 1977	3	ultimo.iii.2022	04-2022	SH	HU	-	HU
*Psilonotusadamas* Walker, 1834	*Semudobiatarda* Roskam, 1977	3	12.iii.2019	iii.2019	SH	PL	-	PL
*Psilonotusadamas* Walker, 1834	*Semudobia* sp. (*betulae* / *tarda*)	2	13.iii.2020	iv.2020	SH	DK	-	DK
*Systasisencyrtoides* Walker, 1834	*Dasineuracrataegi* (Winnertz, 1853)	3	08.viii.2017	viii.2017	SH	DK	N	-
*Toxeumafuscicorne* Walker, 1833	*Dasineurasaxifragae* (Kieffer, 1891)	4B	01.vi.2020	iii.2021	HHB	DK	FCH	DK
Trichomalopsiscf.caricicola (Graham, 1969)	*Planetellagranifex* (Kieffer, 1898)	2	17.iii.2019	v.2019	HHB	DK	FH	DK
**Torymidae**								
*Torymusabbreviatus* Boheman, 1834	*Lasiopterarubi* (Schrank, 1803)	3	18.xii.2015	xii.2015	KA	DK	-	DK
*Torymusanthobiae* Ruschka, 1921	*Contariniaanthobia* (F. Löw, 1877)	3	07.vi.2016	vi.2026	SH	DK	-	DK
*Torymusarundinis* (Walker, 1833)	*Lasiopteraarundinis* Schiner, 1854	NA	02.iv.2018	iv.2018	LKT	DK	-	-
*Torymusarundinis* (Walker, 1833)	*Lasiopteraarundinis* Schiner, 1854	2	07.iv.2014	iv.2014	SH	DK	-	-
*Torymusarundinis* (Walker, 1833)	*Lasiopteraarundinis* Schiner, 1854	2	07.v.2017	v.2017	SH	DK	-	-
*Torymusarundinis* (Walker, 1833)	*Giraudiellainclusa* (Frauenfeld, 1862)	2	12.iii.2021	iv.2021	HHB	DK	-	-
*Torymusarundinis* (Walker, 1833)	*Lasiopteraarundinis* Schiner, 1854	2	17.iv.2014	iv.2014	SH	DK	-	-
*Torymuschloromerus* (Walker, 1833)	Geocrypta campanulae (Müller, 1871)	1A	11.viii.2022	viii.2022	HHB	DK	N	-
*Torymusconfinis* (Walker, 1833)	*Dasineuraurticae* (Perris, 1840)	3	21.xi.2015	xii.2015	SH	DK	-	-
*Torymuscultriventris* Ratzeburg, 1844	*Mikiolafagi* (Hartig, 1839)	3	13.ii.2020	ii.2020	SH	DK	-	-
*Torymuscultriventris* Ratzeburg, 1844	*Mikiolafagi* (Hartig, 1839)	3	20.xi.2015	xii.2015	SH	DK	-	-
*Torymuscultriventris* Ratzeburg, 1844	*Mikiolafagi* (Hartig, 1839)	3	22.ii.2020	iii.2020	SH	DK	-	-
Torymussp. nrcurtisi Graham & Gijswijt	*Kiefferiapericarpiicola* (Bremi, 1847)	2	23.viii.2018	viii.2018	HHB	PL	-	-
*Torymuseglanteriae* Mayr, 1874	*Contariniatiliarum* (Kieffer, 1890)	3	11.vii.2017	vii.2017	SH	DK	-	-
*Torymusfilipendulae* Graham & Gijswijt, 1998	*Dasineuraulmaria* (Bremi, 1847)	3	24.x.2016	xi.2016	SH	DK	-	DK
Torymuscf.filipendulae Graham & Gijswijt, 1998*^6^	*Dasineura* sp. A sensu Harris (2010)	2	20.vi.2020	vii.2020	HHB	DK	FP	-
*Torymusfractiosus* Graham and Gijswijt, 1998	*Dasineurarosae* (Bremi, 1847)	3	02.ix.2020	ix.2020	SH	DK	-	DK
*Torymusfuscicornis* (Walker, 1833)	*Semudobiabetulae* (Winnertz, 1853)	2	03.xii.2015	i.2016	SH	DK	-	-
*Torymusfuscicornis* (Walker, 1833)	*Semudobiabetulae* (Winnertz, 1853)	3	12.iii.2019	iii.2019	SH	PL	-	-
*Torymusfuscicornis* (Walker, 1833)	*Semudobia* sp. (*betulae* / *tarda*)	2	13.iii.2020	iv.2020	SH	DK	-	-
*Torymusfuscicornis* (Walker, 1833)	*Semudobiabetulae* (Winnertz, 1853)	NA	13.xii.2015	i.2016	LKT	DK	-	-
*Torymusfuscicornis* (Walker, 1833)	*Semudobiabetulae* (Winnertz, 1853)	2	17.xii.2015	i.2016	KA	DK	-	-
*Torymusfuscicornis* (Walker, 1833)	*Semudobiabetulae* (Winnertz, 1853)	2	30.xii.2015	i.2016	KA	DK	-	-
*Torymusgaleobdolonis* Graham & Gijswijt, 1998	*Dasineurastrumosa* (Bremi, 1847)	2	23.iv.2022	iv.2022	HHB	DK	-	DK
*Torymusgalii* Boheman, 1834	*Geocryptagalii* (Loew, 1850)	3	06.viii.2020	viii.2020	SH	DK	-	-
*Torymusgalii* Boheman, 1834	*Geocryptagalii* (Loew, 1850)	1A	14.viii.2020	viii.2020	HHB	DK	-	-
*Torymusheyeri* Wachtl, 1883	*Piceacecisabietiperda* (Henschel, 1880)	3	13.ii.2016	v.2016	SH	DK	-	DK
*Torymusjuniperi* (Linnaeus, 1758)	*Oligotrophus* sp.	3	24.viii.2018	xii.2018	SH	PL	-	-
*Torymusmicrostigma* (Walker, 1833)	*Lasiopterarubi* (Schrank, 1803)	3	10.xii.2015	i.2016	KA	DK	N	
*Torymusmicrostigma* (Walker, 1833)	*Putoniellapruni* (Kaltenbach, 1872)	4B	13.vi.2021	iv.2022	HHB	DK	-	-
*Torymusnitidulus* (Walker, 1833)	*Semudobiatarda* Roskam, 1977	2	03.xii.2015	i.2016	SH	DK	-	-
*Torymusnitidulus* (Walker, 1833)	*Semudobiabetulae* (Winnertz, 1853)	3	12.iii.2019	iii.2019	SH	PL	-	-
Torymussp. nrpartitus Graham & Gijswijt, 1998	*Rabdophagadubiosa* Kieffer, 1913	2	17.vii.2014	NA	HHB	DK	FH	DK
*Torymus* sp. nr *T.partitus*/*T.wachtliellae*	*Dasineuralotharingiae* (Kieffer, 1888)	2	26.ix.2015	x.2015	HHB	DK	-	-
*Torymuspersicariae* Mayr, 1874	*Wachtliellapersicariae* (Linnaeus, 1767)	2	09.viii.2022	viii.2022	HHB	DK	-	-
*Torymuspersicariae* Mayr, 1874	*Wachtliellapersicariae* (Linnaeus, 1767)	3	12.viii.2017	viii.2017	SH	DK	-	-
*Torymuspersicariae* Mayr, 1874	*Wachtliellapersicariae* (Linnaeus, 1767)	2	14.vii.2022	vii.2022	HHB	DK	-	-
*Torymusrosariae* Graham and Gijswijt, 1998	*Rabdophagarosaria* (Loew, 1850)	3	03.2021	iv.2021	PB	DK	-	-
*Torymusrosariae* Graham and Gijswijt, 1998	*Rabdophagarosaria* (Loew, 1850)	NA	01.iv.2020	iv.2020	KH	DK	-	-
*Torymusrosariae* Graham and Gijswijt, 1998	*Rabdophagarosaria* (Loew, 1850)	3	06.i.2019	i.2019	SH	DK	-	-
*Torymusrosariae* Graham and Gijswijt, 1998	*Rabdophagarosaria* (Loew, 1850)	3	13.iii.2015	iv.2015	SH	DK	-	DK
*Torymusrosariae* Graham and Gijswijt, 1998	*Rabdophagasalicis* (Schrank, 1803)	NA	14.iv.2020	iv.2020	KH	DK	N	-
*Torymusrosariae* Graham and Gijswijt, 1998	*Rabdophagarosaria* (Loew, 1850)	3	17.xii.2015	i.2016	KA	DK	-	-
*Torymusrosariae* Graham and Gijswijt, 1998	*Rabdophagarosaria* (Loew, 1850)	3	17.xii.2015	i.2016	KA	DK	-	-
*Torymusrosariae* Graham and Gijswijt, 1998	*Rabdophagarosaria* (Loew, 1850)	3	18.xii.2015	i.2016	KA	DK	-	-
*Torymusrosariae* Graham and Gijswijt, 1998	*Rabdophagarosaria* (Loew, 1850)	3	18.xii.2015	i.2016	KA	DK	-	-
*Torymusrubi* (Schrank, 1781)	*Lasiopterarubi* (Schrank, 1803)	3	09.xii.2015	xii.2015	KA	DK	-	-
*Torymusrubi* (Schrank, 1781)	*Lasiopterarubi* (Schrank, 1803)	3	10.xii.2015	xii.2015	KA	DK	-	-
*Torymusrubi* (Schrank, 1781)	*Lasiopterarubi* (Schrank, 1803)	3	11.xii.2015	xii.2015	KA	DK	-	-
*Torymusrubi* (Schrank, 1781)	*Lasiopterarubi* (Schrank, 1803)	3	11.xii.2015	xii.2015	KA	DK	-	-
*Torymusrubi* (Schrank, 1781)	*Lasiopterarubi* (Schrank, 1803)	3	15.xii.2015	xii.2015	KA	DK	-	-
*Torymusrubi* (Schrank, 1781)	*Lasiopterarubi* (Schrank, 1803)	3	17.xii.2015	xii.2015	KA	DK	-	-
*Torymusrubi* (Schrank, 1781)	*Lasiopterarubi* (Schrank, 1803)	3	18.xii.2015	xii.2015	KA	DK	-	-
*Torymusruschkai* (Hoffmeyer, 1929)	*Rhopalomyiaartemisiae* (Bouché, 1834)	2	14.x.2016	i.2016	MB	DK	-	-
*Torymusruschkai* (Hoffmeyer, 1929)	*Rhopalomyiaartemisiae* (Bouché, 1834)	2	17.xii.2016	i.2016	MB	DK	-	-
*Torymusruschkai* (Hoffmeyer, 1929)	*Rhopalomyiaartemisiae* (Bouché, 1834)	2	22.xii.2016	i.2016	MB	DK	-	-
*Torymusruschkai* (Hoffmeyer, 1929)	*Rhopalomyiaartemisiae* (Bouché, 1834)	2	25.x.2016	i.2016	MB	DK	-	-
*Torymusruschkai* (Hoffmeyer, 1929)	*Rhopalomyiaartemisiae* (Bouché, 1834)	2	31.viii.2016	NA	MB	DK	-	-
*Torymussalicis* Graham, 1994	*Iteomyiamajor* (Kieffer, 1889)	3	20.x.2020	xi.2020	SH	DK	FH	DK
*Torymustanaceticola* Ruschka, 1921	*Rhopalomyiatanaceticola* (Karsch, 1879)	3	03.v.2016	v.2016	SH	DK	-	DK
*Torymustanaceticola* Ruschka, 1921	*Rhopalomyiatanaceticola* (Karsch, 1879)	3	11.ix.2016	ix.2016	SH	DK	-	-
*Torymustanaceticola* Ruschka, 1921	*Rhopalomyiatanaceticola* (Karsch, 1879)	3	20.viii.2017	ix.2017	SH	DK	-	-
*Torymustanaceticola* Ruschka, 1921	*Rhopalomyiatanaceticola* (Karsch, 1879)	3	20.x.2016	xi.2016	SH	DK	-	-
*Torymustanaceticola* Ruschka, 1921	*Rhopalomyiatanaceticola* (Karsch, 1879)	3	22.viii.2017	ix.2017	KN	DK	-	-
*Torymustanaceticola* Ruschka, 1921	*Rhopalomyiatanaceticola* (Karsch, 1879)	3	24.ix.2016	x.2016	SH	DK	-	-
*Torymustanaceticola* Ruschka, 1921	*Rhopalomyiatanaceticola* (Karsch, 1879)	3	30.ix.2020	xii.2020	SH	DK	-	-
*Torymustipulariarum* Zetterstedt, 1838	*Rabdophagasalicis* (Schrank, 1803)	3	08.iii.2021	iv.2021	SH	DK	-	-
*Torymustipulariarum* Zetterstedt, 1838	*Rabdophagasalicis* (Schrank, 1803)	3	17.xii.2015	i.2016	SH	DK	-	-
*Torymustipulariarum* Zetterstedt, 1838	*Rabdophagasalicis* (Schrank, 1803)	NA	19.xi.2017	xii.2017	LKT	DK	-	-
*Torymusveronicae* Ruschka, 1921	*Jaapiellaveronicae* (Vallot, 1827)	1A	13.xii.2015	xii.2015	LKT	DK	-	-
*Torymuswachtliellae* Graham and Gijswijt, 1998	*Dasineurarosae* (Bremi, 1847)	3	13.viii.2022	viii.2022	SH	DK	-	DK
*Torymus* sp. A	*Contariniatiliarum* (Kieffer, 1890)	5	04.vii.2020	NA	BWP	DK	-	-
*Torymus* sp. B	*Dasineuraurticae* (Perris, 1840)	5	08.x.2016	NA	SH	DK	-	-
*Torymus* sp. C	*Geocryptagalii* (Loew, 1850)	5	06.viii.2020	NA	SH	DK	-	-
*Torymus* sp. C	*Geocryptagalii* (Loew, 1850)	5	30.vi.2016	NA	SH	DK	-	-
*Torymus* sp. D	*Rabdophagarosaria* (Loew, 1850)	5	29.iii.2019	NA	SH	DK	-	-
*Torymus* sp. E	*Rabdophagasalicis* (Schrank, 1803)	5	23.x.2021	NA	BK	DK	-	-

## Footnotes

*1 Agrees with paratypes from Rostherne and Wychwood (Graham 1969).

*2 Compared with Aprostocetus emesa det. Marcus Graham in making identification.

*3 Males run to M. decipiens Graham in Graham (1969), but M. decipiens Graham has been synonymised under M. penetrans by Mitroiu (2010); Graham described only the male of M. decipiens.

*4 With the reservation that the single female has less slender antennae than expected for P. oeclus.

*5 Agrees with specimens mentioned in Askew and Harris (2007), which were identified by Marcus Graham.

*6 Determination queried because mesoscutal sculpture and gaster shape differ from paratype and specimens reared from Dasineura ulmariae from Fredricia; probably an aberrant specimen.

*7 The legs of the single specimen are darker than Al Khatib et al. (2014) describes for E. tremulae, but otherwise it agrees quite well with the original description.

*8 Specimen in poor condition.

*9 Certainly not Synopeasinerme, which emerged from the same collection in 2022.

## References

[B11198152] Al Khatib Fadel, Fusu Lucian, Cruaud Astrid, Gibson Gary, Borowiec Nicolas, Rasplus Jean-Yves, Ris Nicolas, Delvare Gérard (2014). An integrative approach to species discrimination in the *Eupelmusurozonus* complex (Hymenoptera, Eupelmidae), with the description of 11 new species from the Western Palaearctic. Systematic Entomology.

[B10985725] Askew R. R., Price Peter W. (1975). Evolutionary strategies of parasitic insects and mites.

[B10985747] Askew R. R., Shaw M. R., Waage Jeff, Greathead David J. (1986). Insect Parasitoids: 13th symposium of the Royal Entomological Society of London, 18-19 September 1985 at the Department of Physics Lecture Theatre, Imperial College, London.

[B10985738] Askew R. R., Harris K. M. (2007). Chalcidoidea (Hymenoptera) reared from some gall-inducing Cecidomyiidae (Diptera). Entomologist's Monthly Magazine.

[B10985760] Buhl P. N., Jørgensen J. (2010). Notes on species of Ceraphronidae and Platygastridae (Hymenoptera) reared from Cecidomyiidae (Diptera) in Denmark. Entomologiske Meddelelser.

[B10985769] Chimeno Caroline, Hausmann Axel, Schmidt Stefan, Raupach Michael J., Doczkal Dieter, Baranov Viktor, Hübner Jeremy, Höcherl Amelie, Albrecht Rosa, Jaschhof Mathias, Haszprunar Gerhard, Hebert Paul D. N. (2022). Peering into the darkness: DNA barcoding reveals surprisingly high diversity of unknown species of Diptera (Insecta) in Germany. Insects.

[B11014786] Dorchin N., Harris K. M., Stireman J. O. (2019). Phylogeny of the gall midges (Diptera, Cecidomyiidae, Cecidomyiinae): Systematics, evolution of feeding modes and diversification rates. Molecular Phylogenetics and Evolution.

[B10985786] Espírito-Santo M. M., Fernandes G. Wilson (2007). How many species of gall-inducing insects are there on earth, and where are they?. Annals of the Entomological society of America.

[B10985795] Gaüzère Pierre, O’Connor Louise, Botella Christophe, Poggiato Giovanni, Münkemüller Tamara, Pollock Laura J., Brose Ulrich, Maiorano Luigi, Harfoot Michael, Thuiller Wilfried (2022). The diversity of biotic interactions complements functional and phylogenetic facets of biodiversity. Current Biology.

[B11198059] Graham Marcus William Robert de Vere (1969). The Pteromalidae of Northwestern Europe (Hymenoptera: Chalcidoidea).

[B10985819] Hawkins Bradford A. (1994). Pattern and Process in Host-Parasitoid Interactions. https://www.cambridge.org/core/books/pattern-and-process-in-hostparasitoid-interactions/C3FFD194217077407A996380A34236D4.

[B10985810] Hawkins B. A., Gagné R. J. (1989). Determinants of assemblage size for the parasitoids of Cecidomyiidae (Diptera). Oecologia.

[B10985827] Hebert Paul D. N., Ratnasingham Sujeevan, Zakharov Evgeny V., Telfer Angela C., Levesque-Beaudin Valerie, Milton Megan A., Pedersen Stephanie, Jannetta Paul, deWaard Jeremy R. (2016). Counting animal species with DNA barcodes: Canadian insects. Philosophical Transactions of the Royal Society B: Biological Sciences.

[B10985841] Hortal Joaquín, de Bello Francesco, Diniz-Filho José Alexandre F., Lewinsohn Thomas M., Lobo Jorge M., Ladle Richard J. (2015). Seven shortfalls that beset large-scale knowledge of biodiversity. Annual Review of Ecology, Evolution, and Systematics.

[B11014819] Jennings M. T. (2021). Tetrastichinae (Hymenoptera: Chalcidoidea, Eulophidae) reared from some gall-inducing Cecidomyiidae (Diptera) in Britain. Entomologist's Monthly Magazine.

[B11198014] Kozlov M. A. (1978). Identification of the insects of the European part of the USSR, Vol. 3: Hymenoptera, superfamily Proctotrupoidea, 2. Part: Platygastridae.

[B11198165] Mitroiu Mircea-Dan (2010). Revision of the Palearctic species of *Macroglenes* Westwood (Hymenoptera: Pteromalidae). Zootaxa, , 1–34. Zootaxa.

[B10985852] Roskam J. C. (2013). Biosystematics of insects living in female birch catkins. V. Chalcidoid ectoparasitoids of the genera *Torymus* Dalman, *Aprostocetus* Westwood, *Psilonotus* Walker and *Eupelmus* Dalman (Hymenoptera, Chalcidoidea). Tijdschrift voor Entomologie.

[B10985861] Skuhravá Marcela, Thuróczy Csaba (2007). Parasitic Hymenoptera reared from galls of Cecidomyiidae (Diptera) in Europe. Acta Zoologica Universitatis Comenianae.

[B10985870] Srivathsan Amrita, Ang Yuchen, Heraty John M., Hwang Wei Song, Jusoh Wan F. A., Kutty Sujatha Narayanan, Puniamoorthy Jayanthi, Yeo Darren, Roslin Tomas, Meier Rudolf (2023). Convergence of dominance and neglect in flying insect diversity. Nature Ecology & Evolution.

[B11198022] Szelényi G. von (1938). Über Paläarctische Scelioniden. I. Zur Systematik der Gattung *Inostemma* Walk. Annales Historico-Naturales Musei Nationalis Hungarici.

[B11014836] Tokuda M., Yukawa J., Yukawa J., Tokuda M. (2021). Biology of gall midges: Evolution, ecology, and biological interactions.

[B10985885] Tscharntke T., Abraham R., Vidal S. (1991). Larval characteristics and life-history traits of the parasitoids attacking *Giraudiellainclusa* Fr. (Dipt., Cecidomyiidae). Journal of Applied Entomology.

[B11015079] Tudor Constanţa, Neacşu Petre (1983). Contribuţii la cunoaşterea calcidoidelor parazite pe cecidomiide galigene. Studii şi Cercetări de Biologie - Biologie Animală.

[B11198031] Vlug H. J. (1985). The types of Platygastridae (Hymenoptera, Scelionoidea) described by Haliday and Walker and preserved in the National Museum of Ireland and in the British Museum (Natural History). 2. Keys to species, redescriptions, synonymy. Tijdschrift voor Entomologie.

[B10985894] Yukawa Junichi, Matsuo Kazunori, Fujii Tomohisa, Yukawa Junichi, Tokuda Makoto (2021). Biology of gall midges: Evolution, ecology, and biological interactions.

